# Comparison in Bioactive Compounds and Antioxidant Activity of *Cheonggukjang* Containing Mountain-Cultivated Ginseng Using Two *Bacillus* Genus

**DOI:** 10.3390/foods13193155

**Published:** 2024-10-03

**Authors:** Jina Seong, Hee Yul Lee, Jong Bin Jeong, Du Yong Cho, Da Hyun Kim, Ji Ho Lee, Ga Young Lee, Mu Yeun Jang, Jin Hwan Lee, Kye Man Cho

**Affiliations:** 1 Department of GreenBio Science and Agri-Food Bio Convergence Institute, Gyeongsang National Univesity, Jinju 52725, Republic of Koreagmldbf99@gnu.ac.kr (H.Y.L.);; 2 Department of Life Resource Industry, Dong-A University, 37, Nakdong-daero 550 beon-gil, Saha-gu, Busan 49315, Republic of Korea

**Keywords:** *Bacillus*, *cheonggukjang*, cocktail starters, mountain-cultivated ginseng, nutrients, antioxidant

## Abstract

In this study, the nutrients, phytochemicals (including isoflavone and ginsenoside derivatives), and antioxidant activities of *cheonggukjang* with different ratios (0%, 2.5%, 5%, and 10%) of mountain-cultivated ginseng (MCG) were compared and analyzed using microorganisms isolated from traditional *cheonggukjang*. The IDCK 30 and IDCK 40 strains were confirmed as *Bacillus licheniformis* and *Bacillus subtilis*, respectively, based on morphological, biological, biochemical, and molecular genetic identification, as well as cell wall fatty acid composition. The contents of amino acids and fatty acids showed no significant difference in relation to the ratio of MCG. After fermentation, isoflavone glycoside (such as daidzin, glycitin, and genistin) contents decreased, while aglycone (daidzein, glycitein, and genistein) contents increased. However, total ginsenoside contents were higher according to the ratio of MCG. After fermentation, ginsenoside Rg2, F2, and protopanaxadiol contents of *cheonggukjang* decreased. Conversely, ginsenoside Rg3 (2.5%: 56.51 → 89.43 μg/g, 5.0%: 65.56 → 94.71 μg/g, and 10%: 96.05 → 166.90 μg/g) and compound K (2.5%: 28.54 → 69.43 μg/g, 5.0%: 41.63 → 150.72 μg/g, and 10%: 96.23 → 231.33 μg/g) increased. The total phenolic and total flavonoid contents were higher with increasing ratios of MCG and fermentation (fermented *cheonggukjang* with 10% MCG: 13.60 GAE and 1.87 RE mg/g). Additionally, radical scavenging activities and ferric reducing/antioxidant power were significantly increased in fermented *cheonggukjang*. This study demonstrates that the quality of *cheonggukjang* improved, and *cheonggukjang* with MCG as natural antioxidants may be useful in food and pharmaceutical applications.

## 1. Introduction

Ginseng (*Panax ginseng* C.A Meyer) is classified into artificially cultivated ginseng, mountain wild ginseng, and mountain-cultivated ginseng (MCG) [1]. Recently, interest in MCG has increased alongside growing demand for healthy, functional foods. MCG is less expensive and has higher production rates than wild ginseng while maintaining high pharmacological activity and ginsenoside content, making it a viable alternative to wild ginseng [2]. Tran et al. [3] conducted a comparative study on the anticancer effects of cultivated ginseng, wild ginseng, and MCG, reporting that MCG showed higher effects and contents of physiologically active substances, phenolic components, and free amino acids compared to cultivated ginseng. Known effects of ginseng include antioxidant properties [4], liver toxicity reduction [5], blood lipid improvement [6], angiogenesis promotion [7], anti-inflammatory effects [8], and immune enhancement [9]. MCG contains comparable levels of ginsenosides to regular ginseng. However, there are fewer processing products using MCG compared to ginseng.

Soybean-based foods are generally categorized into processed products (tofu and soymilk) and fermented foods (soybean paste, soy sauce, and *cheonggukjang*) produced using microorganisms [10]. *Cheonggukjang* is rich in essential nutrients like protein, carbohydrates, and fat, and contains numerous physiologically active substances, including isoflavones [11]. During fermentation, the proteins, carbohydrates, and fats in *cheonggukjang* are broken down into easily digestible forms, increasing their absorption rate [12]. Quality indicators such as taste, aroma, texture, color, and functionality of *cheonggukjang* are influenced by raw materials, fermentation conditions, and fermenting microorganisms. Notably, microorganisms play the most crucial role in developing the taste and aroma of *cheonggukjang* [13]. Research on *cheonggukjang* microorganisms has focused on using single or cocktail cultures of *Bacillus* species, including *Bacillus subtilis*, *Bacillus licheniformis*, and *Bacillus megaterium*. The demand for *cheonggukjang* is growing due to its various health benefits, such as blood clot dissolution, improved blood pressure and lipid metabolism, anticancer effects, and antioxidant properties [14]. To enhance its functionality and quality, research on the production of *cheonggukjang* with garlic [15], *deodeok* [16], red ginseng, *Angelica gigas*, and *Rehmanniae radix* [17] has been reported.

Therefore, it was attempted to produce *cheonggukjang* with added MCG not only to increase its usability, but also to enhance its functionality and quality. First, suitable starters for fermenting MCG-added *cheonggukjang* were selected, and *cheonggukjang* with MCG was produced using the selected starter. Finally, the physicochemical properties, nutritional components, phytochemical contents, and antioxidant activities of *cheonggukjang* containing MCG were analyzed and compared to determine the optimal MCG ratio for *cheonggukjang*.

## 2. Materials and Methods

### 2.1. MCG and Chemicals

The traditional *cheonggukjang* (Indang *cheonggukjang:* IDCK) was obtained from the Hamyang-gun Urban Regeneration Center in March 2020. MCG was purchased and used from Ginseng-Bio Co. Ltd. and grown in the Baekjeon-myeon, Hamyang-gun in 2020 (GPS coordinates: 35.575153, 127.613122). Soybeans grown in Hadong-gun in 2022 were stored in the laboratory. *Cheonggukjang* was produced through natural fermentation using environmental microorganisms following traditional methods. Six standard isoflavone compounds (such as daidzein, genistein, malonylgenistin, malonyldaidzin, daidzein, and genistein) were purchased from Sigma Chemical Co. (St. Louis, MO, USA). Ginsenoside standards (including compound K, F1, F2, F3, F5, Rb1, Rb2, Rb3, Rc, Rd, Rd2, Re, Rf, Rg1, Rg2, Rg3, Rh1, Rh2, Ro, protopanaxadiol, and protopanaxtriol) were obtained from KOC Biotech (Daejeon, Korea). Folin–Cicalteu phenol reagent, 2,2-diphenyl-1-picrylhydrazyl (DPPH), 2,2′-Azinobis (3-ethylbenzothiazoline-6-sulfonic acid), diammonium salt (ABTS), 2,4,5-tri(2-pyridyl)-1,3,5-triazine (TPTZ), thiobarbituric acid (TBA), and trichloroacetic acid (TCA) were purchased from Sigma-Aldrich. Methanol, acetonitrile, and water were obtained from J.T.Baker company (Philipsburg, NJ, USA), while other reagents were of analytical grade.

### 2.2. Isolation of Bacillus sp.

First, 10 g of IDCK was suspended in 0.85% NaCl solution, and 0.1 mL aliquots were spread on nutrient agar medium (Difco, Becton Dickinson Co., Sparks, MD, USA) and incubated at 37 °C for 24 h. From the numerous colonies that grew, initial selection was based on morphological similarities to *Bacillus* sp. The selected strains were cultured in tryptic soy broth/agar (TSB/TSA, Difco, Becton Dickinson Co., Sparks, MD, USA) medium, using liquid or solid media as appropriate. Two strains, IDCK 30 and IDCK 40, were ultimately selected. For accurate identification, these strains underwent morphological, biological, biochemical, cell wall fatty acid composition, and molecular genetic analyses.

### 2.3. Identification of Bacillus sp.

#### 2.3.1. Morphological, Physiological, and Biochemical Characteristics

IDCK30 and IDCK40 strains were cultured on tryptic soy agar (TSA, Difco, Detroit, MI, USA) plates to obtain single colonies. Cell morphology was observed using Gram staining and scanning electron microscopy. Physiological characteristics were assessed by evaluating growth at various temperatures (10 °C to 50 °C), pH levels (3–11), and NaCl concentrations (0%–10%). Biochemical properties, including glycolysis, were analyzed using the API50NE kit (bioMérieux, Marcy-l’Étoile, Auvergne-Rhône-Alpes, France).

#### 2.3.2. Cellular Fatty Acids Analysis

Microbial fatty acid compositions were analyzed using gas chromatography (GC-7890, Agilent Technologies, Santa Clara, CA, USA) following pretreatment according to the MIDI Microbial Identification System protocol (Sherlock MIS, MIDI Inc., Newark, DE, USA). The oven temperature started at 170 °C and increased at a rate of 5 °C/min until reaching 260 °C, then increased at a rate of 4 °C/min until reaching 310 °C, where it was maintained for 1 min. Hydrogen was utilized as the carrier gas at a flow rate of 0.5 mL/min [18].

#### 2.3.3. Molecular and Genetic Characteristics

For 16S rRNA, *rec*A, and *gyr*B gene sequencing, genomic DNA was extracted using a G-spin Genomic DNA Purification Kit (iNtRON Biotechnology, Suwon, Republic of Korea). The 16S rRNA, *rec*A, and *gyr*B gene amplification was conducted using the primers indicated in Table S1 [19,20,21]. The polymerase chain reaction (PCR) amplification process consisted of initial denaturation at 95 °C for 5 min, followed by 40 cycles of denaturation at 94 °C for 30 s, annealing at 49 °C for 30 s, and extension at 72 °C for 90 s. After a final extension at 95 °C for 5 min, the reaction was terminated by lowering the temperature to 4 °C. PCR products were purified using the MEGA quick-spin Total Fragment DNA Purification Kit (iNtRON Biotechnology, Suwon, Republic of Korea).

### 2.4. Cheonggukjang Preparation

For single and mixed bacillus fermentation of *cheonggukjang*, 50 g of *Jinyang* soybeans were soaked in water for 12–16 h, then steamed and sterilized at 121 °C for 30 min. The steamed soybeans were inoculated with pre-cultured IDCK10, IDCK30, and IDCK40 bacillus strains and fermented at 35 °C for 5 days. For single bacillus fermentation, 3.0% (*v*/*w*) culture solution was used; for two-strain mixed fermentation, 1.5% (*v*/*w*) of each culture solution was used; and for three-strain mixed fermentation, 1.0% (*v*/*w*) each culture was used. Separately, 90, 95, 97.5, and 100 g of water-soaked *Jinyang* soybeans were mixed with 0% (0 g), 2.5% (2.5 g), 5% (5 g), and 10% (10 g) of dried MCG, respectively, and sterilized at 121 °C for 30 min. These mixtures were inoculated with 2.5% (*v*/*w*) of pre-cultured IDCK30 and IDCK40 bacillus strains and fermented at 35 °C for 5 days (Figure S1). The resulting *cheonggukjang* was dried at 55 °C for 3 days, ground, and stored at −20 °C for experimental use.

### 2.5. Physicochemical Characteristics and Viable Cell Numbers

The pH was measured using a pH meter (Orion Star A211, Thermo Fisher Scientific Inc., Waltham, MA, USA) after stirring 1 g of *cheonggukjang* in 49 mL of distilled water. Acidity was expressed as a percentage of lactic acid by neutralizing the moderately diluted sample to pH 8.31 ± 0.01 with 0.1 N NaOH solution. For reducing sugar analysis, 1 g of sample was added to 10 mL distilled water. Then, the sample suspension was stirred at room temperature for 1 h. Next, 1 mL of the 3,5-dinitrosalicylic acid coloring reagent was added to 0.1 mL of stirred sample and followed by 20 min of reaction at 100 °C. After cooling, the absorbance was measured at 570 nm. The reducing sugar content was calculated using a standard calibration curve prepared with glucose.

The viable cell was mixed with 90 mL of sterile distilled water in 10 g of *cheonggukjang* and diluted step by step to the level of 10^2^ to 10^10^. Then, 0.1 mL was plated on TSA medium and unfolded, and the collected water produced after 24 h of incubation at 37 °C was measured and represented as log CFU/g.

### 2.6. Fatty Acid Analysis

The fatty acid content analysis was performed using the modified method of Vargas-Bello-Pérez et al. [22]. Fatty acid pretreatment involved adding 1.5 mL of 0.5 N NaOH (in methanol) and 0.5 mL of triundecanoin (C_11:0_, 2 mg/mL) to 25 mg of powder sample and heating it at 100 °C for 10 min. Subsequently, 2 mL of boron trifluoride (BF_3_) was added while stirring, and the mixture was heated again for 30 min to facilitate methyl esterification of fatty acids. After the reaction, 1 mL of isooctane was added, vigorously mixed, and left to settle. The isooctane layer was recovered, dehydrated with anhydrous sodium sulfate, filtered through a 0.45 μm membrane filter (Dismic-25CS, Toyoroshikaisha Ltd., Tokyo, Japan), and analyzed using a GC (Nexis GC-2030, Shimadzu Corp., Kyoto, Japan) equipped with an SP-2560 capillary column (100 m × 0.25 mm i.d., 0.2 μm film thickness, Supelco, St. Louis, MO, USA) and a flame ionization detector (FID). A 1 μL injection volume was used, with the injector set at 225 °C and operating in split mode with a split ratio of 200:1. Helium was utilized as the carrier gas at a flow rate of 0.75 mL/min. The oven temperature started at 100 °C, was held for 4 min, and was then increased at a rate of 3 °C/min until reaching 240 °C, where it was maintained for 15 min. The detector temperature was set at 285 °C. Fatty acid contents in the samples were quantified using a standard mixture (CRM47885, Supelco 37 Component FAME Mix, Sigma Aldrich, St. Louis, MO, USA).

### 2.7. Amino Acid Analysis

The amino acid content analysis was performed by adding 4 mL of distilled water to 1 g of powder sample. The mixture was hydrolyzed at 60 °C for 1 h, and then cooled to 4 °C for 2 h after adding 10% 5-sulfosalicylic acid to precipitate proteins. The supernatant was obtained by centrifugation for 3 min and filtered through a 0.45 μm membrane filter. The filtrate was then decompressed and concentrated. The resulting dry matter was reconstituted with 2 mL of lithium buffer (pH 2.2) and filtered again through a 0.45 μm membrane filter before analysis using an amino acid automatic analyzer (L-8900, Hitachi High-Technologies Corp., Tokyo, Japan) [23]. Amino acid contents in the samples were quantified using a standard mixture solution (Type H, Wako Pure Chemical Industries Ltd., Osaka, Japan).

### 2.8. Isoflavone Analysis

Isoflavone content analysis was performed using high-performance liquid chromatography (HPLC) according to the method of Kuligowski et al. [24] with slight modifications. A Lichrophore 100 RP C18 column (4.6 × 250 mm, 5 μm, Merck KGaA, Darmstadt, Germany) was used for the analysis. The mobile phase consisted of HPLC-grade water (solvent A) and acetonitrile (solvent B), both containing 0.2% acetic acid. The mobile phase gradient was as followed: 0 min—100% A, 15 min—90% A, 25 min—80% A, 35 min—75% A, 45 min—65% A, and 50 min—65% A. The sample injection volume was 20 μL, and the flow rate was maintained at 1 mL/min and 30 °C. Isoflavones were detected at UV 254 nm using a diode array detector (DAD). The content of each detected isoflavone was calculated using a standard calibration curve.

### 2.9. Ginsenoside Analysis

The ginsenoside analysis followed the method of Lee et al. [1] using HPLC (Agilent 1200 system, Agilent Technologies Inc., Waldbronn, Germany). First, 1 g of the sample was extracted twice with 20 mL of 70% methanol in a 70 °C water bath for 1 h each time. The extracts were centrifuged, and the supernatant was filtered through a 0.45 μm membrane filter. The combined filtrate was concentrated under reduced pressure at 60 °C, then reconstituted in 2 mL of HPLC-grade water and filtered again through a 0.45 μm membrane filter before analysis. A TSKgel ODS-100Z column (4.6 × 250 mm, 5 μm, Tosoh Corp., Tokyo, Japan) and a DAD detector were used. The flow rate was 1.0 mL/min, and the injection volume was 10 μL. The mobile phase consisted of HPLC-grade water (solvent A) and acetonitrile (solvent B). Detection was performed at 203 nm using the following gradient elution profile (time in minutes –%B): 10 min—19%, 15 min—20%, 30 min—23%, 42 min—30%, 75 min—35%, 80 min—60%, 90 min—80%, and 100 min—80%.

### 2.10. Total Phenolic and Total Flavonoid Contents Analysis

Total phenolic contents (TPC) were analyzed using previously described methods with some modifications [25]. A moderately diluted filtrate extract and a 25% Na_2_CO_3_ solution (0.5 mL each) were combined in a test tube and vortexed for 3 min. Then, 0.25 mL of 2 N Folin–Ciocalteu phenol reagent was added for 1 h before measuring the absorbance at 750 nm using a spectrophotometer. TPC was quantified using a standard calibration curve prepared with gallic acid and expressed as gallic acid equivalents (GAE) mg/g.

Total flavonoid contents (TFC) were determined using the method described by Kim et al. [25] with some modifications. A mixture of 0.5 mL of appropriately diluted filtrate, 1.0 mL of diethylene glycol, and 0.01 mL of 1 N NaOH was prepared in a test tube. The solution was then incubated at 37 °C for 1 h, and to measure the absorbance, it was measured at 420 nm using a spectrophotometer. TFC was quantified using a standard calibration curve prepared with rutin as the reference compound and expressed as rutin equivalents (RE) in mg/g.

### 2.11. Radical Scavenging Activity

The antioxidant activities, including DPPH and ABTS radical scavenging activities and ferric-reducing/antioxidant power (FRAP), were performed according to the method described by Lee et al. [1]. DPPH and ABTS were measured at 525 and 734 nm, respectively, after mixing and reacting the DPPH and ABTS radical solutions with sample extracts. The negative control used an extraction solvent instead of a sample. The difference between the experimental and negative controls was expressed as a percentage (%) using Equation (1):

(1) $$Radical\;scavenging\;activity\left(\%\right)=\left[1-\left(A_{sample}÷A_{control}\right)\right]\times 100$$

*A_sample_*: absorbance of sample, *A_control_*: absorbance of negative control

For FRAP measurements, acetate buffer, TPTZ reagent, and FeCl_3_ solution were preliminarily mixed. Then, 50 μL of extract and 950 μL of FRAP reagent were combined in a test tube and incubated at 37 °C for 15 min. The absorbance was measured at 593 nm using a spectrophotometer (UV-1800 240V, Shimadzu Corp., Kyoto, Japan).

### 2.12. Statistical Analysis

Values were presented as the mean ± standard deviation of pentaplicate determination. Statistical significance between samples was determined using one-way analysis of variance, followed by Duncan’s multiple range test (*p* < 0.05). All analysis were performed using the Statistical Analysis System (SAS) software (version 9.4; SAS Institute, Cary, NC, USA).

## 3. Results and Discussion

### 3.1. Identification of Fermented Soybean Strains with IDCK 30 and IDCK40

**Figure 1:**
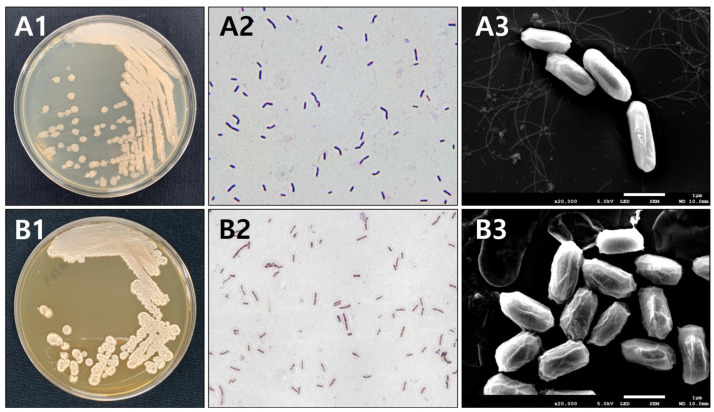
Morphological characteristics of strains with IDCK30 and IDCK40. (**A1**) Colony shape of strain IDCK30 in TSA media; (**A2**) optical microscope of gram-stained strain IDCK30; (**A3**) scanning electron microscopy of strain IDCK30; (**B1**) colony shape of IDCK40 in TSA media; (**B2**) optical microscopy of gram-stained strain IDCK40; and (**B3**) scanning electron microscopy of strain IDCK40.

The identification results of the IDCK30 and IDCK40 strains based on morphological and biochemical characteristics are presented in Figure 1 and Table S2. Both the IDCK30 and IDCK40 strains showed bacillus morphology, were gram-positive, and had flagella and spores. Physiologically, IDCK30 hydrolyzed starch, cellulose, and xylan, while IDCK40 hydrolyzed all six tested substrates. IDCK30 used 18 carbon sources, including glycerol, L-arabinose, ribose, D-glucose, and D-fructose, showing 99.9% similarity to *Bacillus pumilus*. IDCK40 used 17 carbon sources, including L-arabinose, ribose, D-xylose, D-glucose, and D-fructose, demonstrating 99.9% similarity to *B. subtilis*/*amyloliquefaciens*. Optical and scanning electron microscopy revealed that IDCK30 colonies had relatively smooth surfaces, while IDCK40 colonies displayed surface irregularities.

Since the fatty acid composition of the cell wall is a major indicator for classifying and identifying bacteria, it was analyzed using microbial identification GC. Supplementary Table S3 shows the results of analyzing the fatty acid composition of the IDCK30 and IDCK40 strains. Among branched fatty acids, anteiso-C15:0 showed the highest proportion in both IDCK30 (32.15%) and IDCK40 (47.31%) strains.

The analysis results of 16S rRNA, *rec*A, and *gyr*B gene sequence similarity in the fermented strains are shown in Table 1 and Figure S2–S7. For accurate identification, the IDCK30 and IDCK40 16S rRNA, *rec*A, and *gyr*B gene sequences were analyzed. IDCK30 showed 98–99% similarity with *B. licheniformis*. IDCK40 showed 99% similarity with *B. subtilis*. Based on morphological, physiological, cell wall fatty acid composition, biochemical, and molecular genetic characteristics, the IDCK30 strain was identified as *B. licheniformis* and the IDCK40 strain as *B. subtilis*.

Previous studies have reported that *B. subtilis*, *B. licheniformis*, and *Bacillus* sp. are known as major fermentation microbial species of *cheonggukjang* [15,26]. Therefore, it is considered that *B. licheniformis* IDCK 30 and *B. subtilis* IDCK 40 strains are appropriate for producing *cheonggukjang.*

### 3.2. Characteristics of Cheonggukjang with Single and Cocktail Starters

The analysis results of physicochemical properties, viable cell numbers, free amino acids, isoflavones, physiological activity components, and radical scavenging activities in fermented *cheonggukjang* with single and complex starters are presented in Table 2. The pH of *cheonggukjang* with IDCK30 increased, while acidity decreased. Conversely, *cheonggukjang* with IDCK40 and IDCK30 + IDCK40 showed decreased pH and increased acidity. Reducing sugar content decreased in all *cheonggukjang* samples with starters after fermentation, and viable cell numbers ranged from 10.20 to 10.57 log CFU/g. Total free amino acid contents increased during fermentation compared to steamed soybeans (4.31 mg/g), with IDCK40 (120.02 mg/g) showing the highest content, followed by IDCK30 + IDCK40 (94.88 mg/g) and IDCK30 (46.64 mg/g). The total contents of six isoflavones (daidzin, glycitin, genistein, daidzein, glycitein, and genistein) were confirmed with 2154.6 (steam), 1405.1 (IDCK30), 2134.7 (IDCK40), and 1575.3 (IDCK30 + IDCK40) μg/g. Aglycone contents were highest (1014.53 μg/g) in *cheonggukjang* with the IDCK30 + IDCK40 starter. TPC and TFC were highest in *cheonggukjang* with the IDCK40 starter (13.91 and 1.488 mg/g), and DPPH and ABTS radical scavenging activities followed the same trend (57.57 and 49.63%). Based on a comprehensive comparison of physiologically active substances and antioxidant activities, IDCK30 + IDCK40-fermented *cheonggukjang* was deemed superior, leading to a further experiment with a cocktail strain.

Cho et al. [27] reported that the *cheonggukjang* with starter *B. pumilus* HY1 increased the activity of β-glucosidase and esterase, correspondingly reading the TFC, gallic acid, and aglycone contents. Also, the *cheonggukjang* with *B. subtilis* CS90 increased phytochemicals (isoflavones, flavanols, phenolic acids) during fermentation according to an increase in β-glucosidase and esterase activity [28]. Shin et al. [29] reported that fermented *cheonggukjang* by *B. subtilis* CSY191 increased TPC and aglycone, corresponding to antioxidant activities. In our prior research, it was demonstrated that β-glucosidase and esterase produced by *Bacillus* strains converted isoflavone glycosides to aglycones and esterified phenolic acid compounds to free phenolic acids, respectively. This conversion resulted in increased TPC, TFC, and antioxidant activity [27,28,29].

### 3.3. Comparison of Physicochemical Characteristics and Viable Cell Numbers in Cheonggukjang According to MCG Ratio

Table 3 presents the analysis results of the physicochemical characteristics in *cheonggukjang* according to the MCG addition ratio. In the unfermented sample, pH values were low as the MCG concentration increased from 0% to 10% (pH 6.81, 6.76, 6.26, and 6.20), while the acidity values were correspondingly high. After fermentation, the pH values slightly decreased, except for 10% MCG *cheonggukjang* (pH 7.38, 6.66, 6.07, and 6.08), and acidity slightly increased, except for 10% MCG *cheonggukjang* (0.76, 1.05, 1.09, and 1.11%). Reducing sugar notably decreased after fermentation from 28.36–28.24 mg/g (unfermented) to 6.63–10.6 mg/g (fermented). The number of viable cells increased after fermentation from an average level of 5.75 log CFU/g (unfermented) to 8.08–9.20 log CFU/g (fermented). As the MCG ratio increased, the number of viable cells was found to be inversely proportional.

Recent literature has demonstrated that a decrease in pH and an increase in acidity were observed in ginseng and red ginseng after fermentation. Lee et al. [1] reported that pH was decreased and the acidity values were increased during the processing steps of aging and fermentation. Lee et al. [30] reported that pH decreased during food processing from 5.4 (dry MCG) to 4.44 (fermented red MCG), corresponding to an increase in acidity from 0.21 to 0.51 [30]. Our present results are consistent with these previously reported findings [1,30].

### 3.4. Comparison of Free Amino Acid Contents of Cheonggukjang According to MCG Ratio

The analysis results of free amino acid contents in *cheonggukjang* based on MCG addition ratios is presented in Table 4. Amino-type nitrogen, primarily amino acids, is produced from soy proteins via peptides through the action of microbial proteases during *cheonggukjang* fermentation, contributing to its savory taste [31]. In unfermented *cheonggukjang*, total free amino acid contents were slightly higher as MCG levels rose from 0% to 5%, except MCG 10% (536.77, 544.84, 589.43, and 551.85 mg/100 g, respectively). In fermented *cheonggukjang*, free amino acid contents increased more than 20-fold due to various enzymatic actions. Viable cell numbers increased; the highest content was observed in 0% MCG *cheonggukjang*, along with the highest ammonia content, potentially resulting in a strong, putrid odor. Among the increased free amino acids, glutamic acid (54.68 to 65.90 mg/100 g → 1401.07 to 2720.27 mg/100 g), a non-essential amino acid, showed the most significant increase.

Overall, the amino acid amounts in the *cheonggukjang* increased notably during fermentation. Most amino acids showed a substantial increase within the first 12 h of fermentation. Notably, glutamic acid, which is strongly associated with the umami taste in *cheonggukjang*, increased tenfold by the end of fermentation compared to its initial levels [29]. Additionally, various free amino acids increased. Interestingly, ornithine was not detected in unfermented *cheonggukjang*, but in fermented *cheonggukjang*, its content increased with higher ratios of MCG, reaching a maximum of 681.77 mg/100 g in *cheonggukjang* with 10% MCG. Conversely, arginine content decreased in fermented *cheonggukjang* compared to unfermented *cheonggukjang*. This decrease in arginine and increase in ornithine was likely due to the action of arginase produced by microorganisms during fermentation. This observation aligns with previous research suggesting the conversion of arginine to ornithine during fermentation [32]. *B. subtilis*, a major microorganism in fermented foods such as *cheonggukjang,* produces microbial arginase. The gene encoding arginase was isolated from *B. subtilis* 168. Furthermore, functional fermented foods such as *cheonggukjang* have been reported to have enhanced levels of ornithine, and pharmaceutical products have been developed using the key enzyme in arginine degradation and ornithine production [33]. Therefore, compared to the control (0% MCG), fermented *cheonggukjang* with added MCG is considered to have high potential as a food with such functionality.

### 3.5. Comparison of Fatty Acid Contents of Cheonggukjang According to MCG Ratio

The analysis results of fatty acid contents in *cheonggukjang* based on the MCG addition ratio are presented in Table 5. Total fatty acid contents were highest in 2.5% MCG, with unfermented and fermented *cheonggukjang* containing 1482.34 and 1449.01 mg/100 g, respectively. The major fatty acids in all samples were palmitic acid, oleic acid, linoleic acid, and α-linolenic acid. After fermentation, the contents of palmitic acid and behenic acid among saturated fatty acids, elaidic acid, and eicosadienoic acid among unsaturated fatty acids relatively increased. Other fatty acid contents showed minor increases or decreases. The results indicated no significant difference in fatty acid contents based on the MCG addition ratio.

Recently, Cho et al. [34] reported that *Neulchan* soybean cultivars showed palmitic acid (10.33 ± 0.44) and stearic acid (3.61 ± 0.15) after 48 h of fermentation using *B. subtilis* CSY19. Fatty acid contents varied depending on the soybean cultivars. Unsaturated fatty acids, such as linoleic acid, oleic acid, palmitic acid, and linolenic acid, contributed to approximately 80% of fatty acid content.

The soybean cultivar used in this study was *Daewon*, and although different from the previous study, it similarly had contents of palmitic acid, stearic acid, and unsaturated fatty acids. Chung et al. [35] reported that mountain-cultivated ginseng roots had higher fatty acid contents than field-cultivated ginseng or wild mountain ginseng. The most abundant unsaturated fatty acids were linoleic acid, palmitic acid, and oleic acid, in that order. However, unlike previous studies, our study used a whole MCG rather than just the roots. Similarly, unsaturated fatty acid contents in ginseng roots were typically 2.5-fold higher than saturated fatty acid contents. These results suggest that the root part of MCG may contribute significantly to the fatty acid content. Although fermentation influenced fatty acid contents, MCG addition did not. Since fatty acid contents did not increase with MCG addition, the amount of MCG added may not be a key factor in fatty acid production.

### 3.6. Comparison of Isoflavone and Ginsenoside Contents of Cheonggukjang According to MCG Ratio

**Figure 2:**
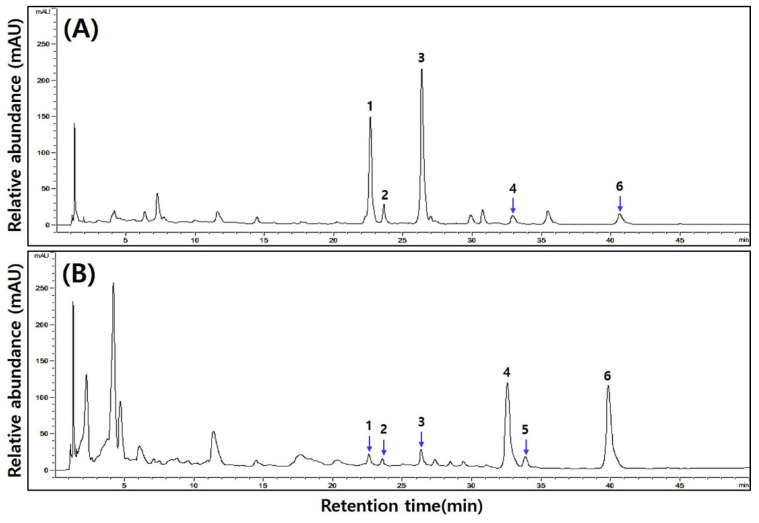
Isoflavone HPLC chromatography of *cheonggukjang* according to the addition ratio of mountain-cultivated ginseng (MCG). (**A**) 10% MCG addition to unfermented *cheonggukjang*. (**B**) 10% MCG addition to fermented *cheonggukjang*. Peak 1, daidzin; peak 2, glycitin; peak 3, genistin; peak 4, daidzein; peak 5, glycitein; peak 6, genistein.

The analysis results of isoflavone contents in *cheonggukjang* according to the MCG addition ratio are shown in Table 6 and Figure 2. Six compounds were detected: daidzin (peak 1), glycitin (peak 2), genistin (peak 3), daidzein (peak 4), glycitein (peak 5), and genistein (peak 6). The contents of glycosides (daidzin, glycitin, and genistin) before fermentation were generally inversely proportional to the ratio of MCG. After fermentation, as the glycoside form decreased, the content of daidzein in the form of aglycones increased. Fermented *cheonggukjang*: 0% (57.65 → 612.53 μg/g) > 2.5% (51.26 → 575.99 μg/g) > 5.0% (48.06 → 545.13 μg/g) > 10% (48.34 → 483.17 μg/g). The total isoflavone content was the highest in 0% MCG both before and after fermentation. The increase in daidzein after fermentation occurred due to the enzymatic hydrolysis of daidzin into its aglycone form by β-glucosidase produced by bacteria. Consequently, the daidzein content showed a similar trend corresponding to the daidzin content before fermentation.

**Figure 3:**
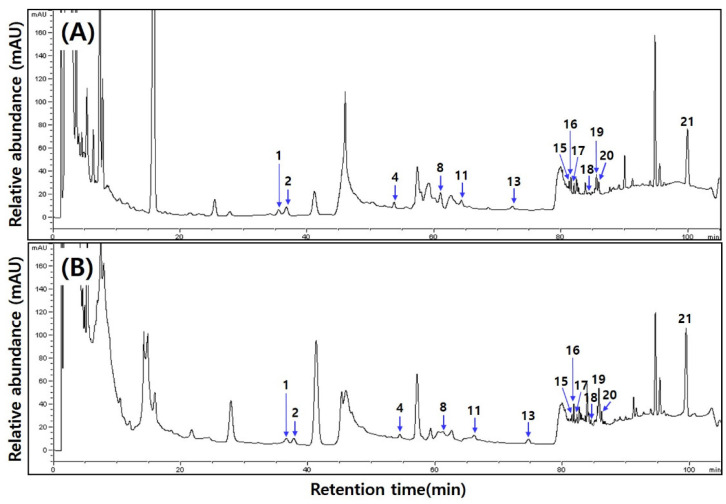
Ginsenoside HPLC chromatography of *cheonggukjang* according to the addition ratio of mountain-cultivated ginseng (MCG). (**A**) 10% MCG addition to unfermented *cheonggukjang*. (**B**) 10% MCG addition to fermented *cheonggukjang*. Peak 1, Rg1; peak 2, Re; peak 3, Ro; peak 4, Rf; peak 5, F5; peak 6, Rb1; peak 7, F3; peak 8, Rg2; peak 9, Rh1; peak 10, Rc; peak 11, Rb2; peak 12, Rb3; peak 13, F1; peak 14, Rd; peak 15, Rd2; peak 16, F2; peak 17, Rg3; peak 18, protopanaxtriol; peak 19, compound K; peak 20, Rh2; peak 21, protopanaxdiol.

Table 6 and Figure 3 show the analysis results of ginsenoside contents in *cheonggukjang* based on MCG addition ratios. Ginsenosides were not detected in the 0% MCG sample before or after fermentation. In MCG-added samples, the ginsenoside content increased with higher MCG concentrations and further decreased after fermentation (2.5%: 1043.97 → 1315.79 μg/g, 5.0%: 1567.11 → 1562.99 μg/g, 10%: 2718.81 → 2480.74 μg/g). Rg2, F2, and protopanaxadiol were the major ginsenosides detected in MCG-added *cheonggukjang* before fermentation. Minor ginsenosides, including Rf, protopanaxtriol, Rg3, and compound K, were also commonly detected. Most of the ginsenosides increased after fermentation, with notable increases in Rg3 (2.5%: 56.51 → 89.43 μg/g, 5.0%: 65.56 → 94.71 μg/g, 10%: 96.05 → 166.90 μg/g) and compound K (2.5%: 28.54 → 69.43 μg/g, 5.0%: 41.63 → 150.72 μg/g, 10%: 96.23 → 231.33 μg/g).

The absorption rate in the aglycone form is higher than in the glycoside form, but the amount of aglycones is small in raw soybeans [36,37]. Therefore, to increase the absorption rate of isoflavones in the body, bioconversion process technology and food processing techniques that convert isoflavones into aglycone form are required [38]. The β-glucosidase, essential in biomass conversion, is often isolated from bacteria or fungi by removal of non-reducing terminal glucosyl residues from saccharides and glycosides [39]. Isoflavones exist predominantly as the glycoside forms rather than aglycone forms in soybeans, but are converted from the glycoside to the aglycone forms by the action of β-glucosidase from *Bacillus* spp. during *cheonggukjang* fermentation [40]. Previous studies have shown that the proportion of aglycones in total isoflavones is markedly higher in *cheonggukjang* fractions compared to steamed soybeans [41]. Cho et al. [28] reviewed changes in pH, β-glucosidase, and esterase activities during *cheonggukjang* fermentation by *B. subtilis* CS90. They revealed that, among various fermentation times, the crude extract of *cheonggukjang*, fermented for 36 h, might be most effective for β-glucosidase and esterase activities. In our study, the increase in β-glucosidase activity during fermentation with starter IDCK30 + 40 suggested that the content of daidzein, an aglycone form, increased in fermented *cheonggukjang* compared to in unfermented *cheonggukjang*. However, the difference in isoflavone contents was not significant according to the MCG ratio. Therefore, it was considered that MCG did not affect β-glucosidase production.

Ginsenoside Rb1, which accounts for 20% of the total ginsenosides, is commonly used as a precursor to produce minor ginsenosides via β-glucosidases [42]. Specifically, ginsenoside Rb1 is converted to Rd by β-D-glucosidase [43]. Our findings align with previous reports showing that fermentation, high temperature, and aging processes increase the concentration of minor ginsenosides Rg2, Rg3, and compound K, which have higher physiological activity [1]. The primary factors affecting the improvement of ginsenoside pharmacological activities include temperature, heating time, extraction solvent, and stability [44]. Fermentation reduces pH value and increases acidity [1]. By reducing the pH, ginsenoside can be easily deglycosylated and dehydrated at the C-20 position of aglycones when heated under mild acidic conditions rather than neutral and basic conditions. Properties of MCG were changed upon fermentation. To be specific, through acid hydrolysis, formic acid promotes the transformation from ginsenoside Re, Rf, and Rg2 into rare ginsenosides F1, Rh1, Rf2, Rf3, Rg6, and F4 [41]. Compound K (CK), not present in native ginseng, is a degradation product of panaxadiol saponins like ginsenoside Rb1 and Rb2 due to intestinal microflora [45,46]. The rare ginsenoside CK, derived from *Panax ginseng*, is a panaxadiol saponin that enhances immune function, has anti-inflammatory properties, and resists skin aging, with high bioavailability and absorption in the human body [1,47]. In this study, the Rd2, F2, Rg3, and CK contents in *cheonggukjang* increased with the proportion of MCG addition, with 10% MCG addition yielding the highest ginsenoside content [48]. Accordingly, it is believed that immune and anti-inflammatory properties can be obtained when consuming *cheonggukjang* with added MCG compared to 0% MCG *cheonggukjang*. In our study, *cheonggukjang* with MCG addition had beneficial effects on the acidity increase, ginsenoside content, and isoflavone levels through the fermentation process. And, through this, the possibility of MCG-added *cheonggukjang* as a functional food was confirmed.

### 3.7. Comparison of TPC and TFC of Cheonggukjang According to MCG Ratio

**Figure 4:**
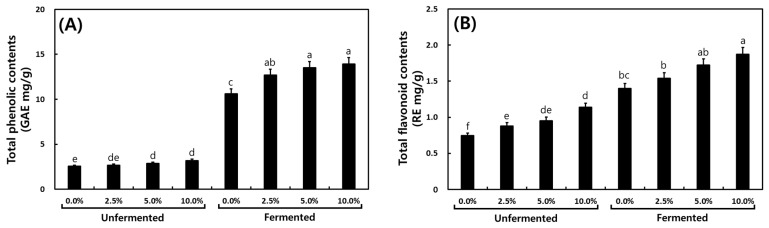
Total phenolic and total flavonoid contents of *cheonggukjang* according to the addition ratio of mountain-cultivated ginseng. (**A**) Total phenolic contents. (**B**) Total flavonoid contents. Different letters above the bars indicate significant difference at *p* < 0.05 (*n = 5*).

Figure 4 illustrates the analysis results of TPC and TFC in *cheonggukjang* relative to the MCG addition ratio. In unfermented *cheonggukjang*, TPC was higher proportionally with the MCG addition (2.55, 2.61, 2.68, and 2.93 GAE mg/g). For fermented *cheonggukjang*, TPC was observed to be highest in the 10% MCG sample at 13.60 GAE mg/g. The TFC results were similar to those of TPC. TFC increased after fermentation, and the 10% MCG *cheonggukjang* showed the highest TFC at 1.87 RE mg/g.

Phenolic acids like gallic acid and its derivatives exhibit antioxidant, antimutagenic, and anticarcinogenic properties. Daily intake is suggested to offer various health benefits, including reducing disease risk [49,50,51]. While numerous studies have explored increasing ingredients through fermented ginseng, research on TPC and TFC in relation to MCG content remains limited. Fermented aging mountain-cultivated ginseng sprout (FAMCGS) demonstrated the highest average TPC and TFC compared to other processes (MCGS: mountain-cultivated ginseng sprout and AMCGS: aging mountain-cultivated ginseng sprout) [1]. A previous study found that soybeans fermented with *B. subtilis* had a total phenol content approximately three times higher than unfermented soybeans [52]. Additionally, fermented soybean products with altered isoflavone and phenolic contents showed stronger antioxidant activity than non-fermented ones [53]. Our results align with previous literature reporting that processed soybean products using thermal and fermentative techniques have higher TPC, TFC, and antioxidant capacities than fresh soybean. These changes also occurred due to varying MCG concentrations. These results were similar to the increase in phenol content in *cheonggukjang* with the addition of garlic [15], red ginseng, *Angelica gigas*, and *Rehmanniae radix* [17], and thus, it was confirmed that MCG contributed to the increase in phenol content in *cheonggukjang*. Consequently, fermenting *cheonggukjang* with MCG may be recommended as a potent food technique for developing nutraceutical agents and functional materials.

### 3.8. Comparison of Antioxidant Activity of Cheonggukjang According to MCG Ratio

**Figure 5:**
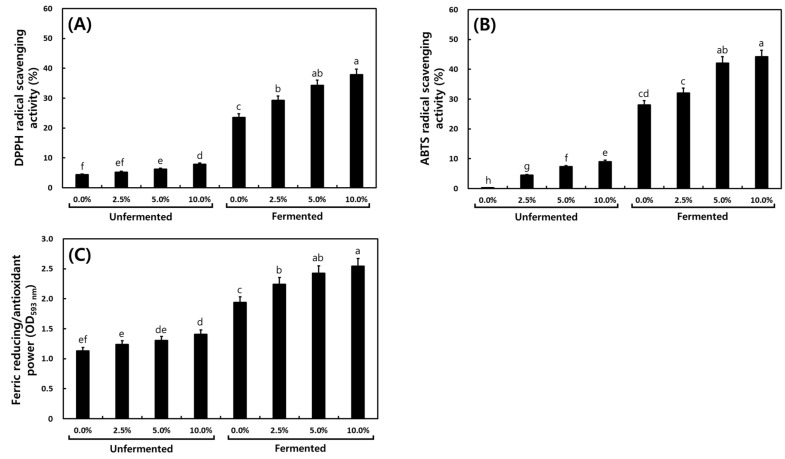
Antioxidant activities of *cheonggukjang* according to the addition ratio of mountain-cultivated ginseng. (**A**) DPPH radical scavenging activity. (**B**) ABTS radical scavenging activity. (**C**) Ferric-reducing/antioxidant power. Different letters above the bars indicate significant differences at *p* < 0.05 (*n = 5*).

The antioxidant activities in *cheonggukjang* according to the MCG addition ratio are shown in Figure 5. DPPH radical scavenging activity increased after fermentation, and the activity increased the MCG ratios correspondingly: 0% MCG (4.39 → 23.59%), 2.5% (5.24 → 29.54%), 5% (6.24 → 34.35%), and 10% (7.90 → 37.86%) (Figure 5A). ABTS radical scavenging activity also increased after fermentation in all samples, from 0.30%, 4.47%, 7.35%, and 9.03% (unfermented) to 28.04%, 32.09%, 42.11%, and 44.22% (fermented), respectively (Figure 5B). FRAP was also increased from 1.23, 1.29, 1.31, and 1.41 OD_593nm_ (unfermented) to 1.94, 2.24, 2.43, and 2.54 OD_593nm_ (fermented), showing a similar trend to radical scavenging activities, but with a slight increase after fermentation compared to DPPH and ABTS radical scavenging activities (Figure 5C). These antioxidant activity results showed similar patterns with the TPC and TFC results.

It is known that the antioxidant effect of MCG depends on the amount added and the processing methods. A similar trend was observed for TPC, indicating that the DPPH radical scavenging activities of MCG and CG could be attributed to their antioxidant and phenolic compounds [54]. Based on the results of antioxidant activities, it is suggested that MCG showed antioxidant properties, especially the effect of fermented 10% MCG *cheonggukjang*, which was stronger than unfermented 10% MCG. Our results were similar to previously published data showing that antioxidant properties showed high increase rates during fermentation processes. Previous research has shown that changes in DPPH radical-scavenging activity during *cheonggukjang* fermentation increased from 54.5% to 96.2% by 60 h [15]. Antioxidant activities of foods, including soybeans, can be influenced by the contents of phenolics and aglycones [15]. Furthermore, the transformation of isoflavone glycosides to aglycones, as well as the increase in TPC, TFC, and antioxidant activities during fermentation, improved the functional properties and bioavailability of *cheonggukjang*. Previous studies on *cheonggukjang* with added garlic [15] and other ingredients [17] have also reported an increase in antioxidant activity due to an increase in phenolic substances, and it was increased with added garlic, red ginseng, *Angelica gigas*, and *Rehmanniae radix* [15,17]. These results also confirmed an increase in antioxidant activity due to the addition of MCG and an increase in the amount added. This suggests that *cheonggukjang* possesses potential as an additive for functional foods and nutraceuticals to reduce oxidative stress [55].

## 4. Conclusions

In this study, strains isolated from *cheonggukjang* in *Indang* town were selected to improve the preference and functional characteristics of *cheonggukjang*. The physiologically active substances, antioxidant activity, etc., of *cheonggukjang* fermented with IDCK 30 and 40, individually or combined, were compared. IDCK 30 + 40 was chosen for fermenting *cheonggukjang* containing MCG. The nutrients, phytochemical properties, and antioxidant activities of *cheonggukjang* with different MCG ratios (0%, 2.5%, 5%, and 10%) were analyzed. Fatty acid contents showed no significant difference across MCG ratios. Total isoflavone content was highest before fermentation, while total aglycone content was highest after fermentation. Total ginsenoside content was undetected in MCG-free samples but increased with MCG addition, decreasing after fermentation (2.5%: 1043.97 → 1315.79 μg/g, 5.0%: 1567.11 → 1562.99 μg/g, 10%: 2718.81 → 2480.74 μg/g). But ginsenoside F2, Rg3, and compound K were increased after fermentation. TPC and TFC contents increased with MCG addition and after fermentation (2.61, 2.68, and 2.93 GAE mg/g). DPPH radical scavenging activity increased after fermentation and with MCG addition: 0% MCG (4.39 → 23.59%), 2.5% (5.24 → 29.54%), 5% (6.24 → 34.35%), and 10% (7.90 → 37.86%). ABTS radical scavenging activity (after fermentation: 28.04 < 32.09 < 42.11 < 44.22%) and FRAP (after fermentation: 1.94 < 2.24 < 2.43 < 2.54 OD_593nm_) also increased. Fermentation significantly enhanced the bioavailability and functional properties of *cheonggukjang* via conversion from isoflavone glycosides to aglycones. Limited research exists on antioxidant activity related to MCG ratios. This study aimed to improve *cheonggukjang* quality and provide foundational data for diversification and value addition of *cheonggukjang* as a natural antioxidant in the food industry.

## Figures and Tables

**Table 1:** 16S rRNA, *rec*A, and *gyr*B gene sequence similarities of strains IDCK30 and IDCK40.

Genes	Isolates	Nearest Relatives ^1^ (Accession No.)	Similarity (%)
16S rRNA	IDCK30	*Bacillus licheniformis* B.licheCEL (LC006127)	99
	IDCK40	*Bacillus subtilis* subsp. *subtilis* 2KL1 (CP032872)	99
*rec*A	IDCK30	*Bacillus licheniformis* P8_B2 (CP045814)	99
	IDCK40	*Bacillus subtilis* P5 (CP045816)	99
*gyr*B	IDCK30	*Bacillus licheniformis* P8_B2 (CP045814)	98
	IDCK40	*Bacillus subtilis* MB9_B6 (CP045818)	99

^1^ Accession number of the nearest relative. If more than one sequence had the same similarity value, only the accession number of the first sequence is given.

**Table 2:** Characteristics of *cheonggukjang* according to single and complex starters.

Index ^1^	Steam	Starters		
		IDCK30	IDCK40	IDCK30 + 40
**Physicochemical Properties**				
pH	6.81 ± 0.03b	7.26 ± 0.02a	6.20 ± 0.06c	6.27 ± 0.06c
Acidity (%, as lactic acid)	0.90 ± 0.01c	0.85 ± 0.01d	1.06 ± 0.01a	1.03 ± 0.01b
Reducing sugar (mg/g)	20.71 ± 0.09a	6.98 ± 0.05d	11.86 ± 0.11b	8.10 ± 0.06c
Viable cell numbers (log CFU/g)	nd ^2^	10.57 ± 0.08a	10.27 ± 0.09b	10.51 ± 0.11a
Free amino acid contents (mg/g)				
Non-essential amino acids	4.31 ± 0.06d	24.61 ± 0.27c	65.06 ± 0.85a	48.62 ± 0.59b
Essential amino acids	0.73 ± 0.01d	22.03 ± 0.25c	54.96 ± 0.65a	46.26 ± 0.86b
Total amino acids	5.04 ± 0.03d	46.64 ± 0.54c	120.02 ± 2.20a	94.88 ± 1.05b
Total isoflavone contents (μg/g)	2154.6 ± 35.6a	1405.1 ± 28.7c	2134.7 ± 40.5a	1575.3 + 29.2b
Daidzin	709.5 ± 14.2b	143.5 ± 2.9d	820.9 ± 14.5a	175.8 ± 3.2c
Glycitin	303.2 ± 4.1b	133.9 ± 2.5d	500.8 ± 10.0a	240.5 ± 4.1c
Genistin	934.4 ± 13.7a	155.2 ± 3.3c	634.3 ± 12.7b	144.5 ± 2.5d
Total glycosides	1947.1 ± 32.0a	432.6 ± 8.5c	1956.0 ± 37.2a	560.8 ± 9.8b
Daidzein	44.9 ± 0.9d	660.8 ± 13.9a	78.1 ± 1.6c	517.2 ± 10.1bc
Glycitein	117.0 ± 2.1a	88.1 ± 1.5b	28.3 ± 0.5c	86.2 ± 1.5b
Genistein	45.6 ± 0.6d	223.6 ± 4.8b	72.3 ± 1.2c	411.1 ± 7.8a
Total aglycones	207.5 ± 3.6c	972.5 ± 20.2b	178.7 ± 3.3d	1014.5 ± 19.4a
Total phenolic contents (mg/g)	2.58 ± 0.02d	10.79 ± 0.08c	13.91 ± 0.11a	13.31 ± 0.11b
Total flavonoid contents (mg/g)	0.487 ± 0.004d	0.787 ± 0.009c	1.488 ± 0.021a	1.374 ± 0.011b
Radical scavenging activity (%)				
DPPH (1 mg/mL)	7.81 ± 0.05d	33.57 ± 0.19c	57.57 ± 0.65a	52.35 ± 0.50b
ABTS (0.5 mg/mL)	10.78 ± 0.09d	33.44 ± 0.23c	49.63 ± 0.48a	48.07±0.49b

^1^ All values are presented as the mean ± standard deviation of pentaplicate determination. Means with different letters within a row are significantly different between samples for the same index (*p* < 0.05).^2^ nd, not detected.

**Table 3:** Phytochemical characteristics of *cheonggukjang* according to the addition ratio of mountain-cultivated ginseng.

Index ^1^	Addition Ratio of Mountain-Cultivated Ginseng (%)							
	Unfermented *Chenoggukjang*				Fermented *Chenoggukjang*			
	0	2.5	5.0	10	0	2.5	5.0	10
pH	6.81 ± 0.08b	6.76 ± 0.07b	6.26 ± 0.10d	6.20 ± 0.07d	7.38 ± 0.09a	6.66 ± 0.08bc	6.07 ± 0.08e	6.08 ± 0.07e
Acidity (%, as lactic acid)	0.90 ± 0.01d	0.92 ± 0.01d	1.05 ± 0.01b	1.06 ± 0.01b	0.76 ± 0.01c	1.05 ± 0.01b	1.09 ± 0.01a	1.11 ± 0.01a
Reducing sugars (mg/g)	28.36 ± 0.31b	32.87 ± 0.43a	28.38 ± 0.38b	28.24 ± 0.28b	6.63 ± 0.07f	8.94 ± 0.10e	9.31 ± 0.13d	10.06 ± 0.10c
Viable cell numbers (log CFU/g)	5.81 ± 0.07f	5.83 ± 0.05e	5.76 ± 0.06f	5.61 ± 0.06g	9.56 ± 0.10a	9.33 ± 0.11b	9.12 ± 0.12c	8.80 ± 0.12d

^1^ All values are presented as the mean ± standard deviation of pentaplicate determination. Means with different letters within a row are significantly different between samples for the same index (*p* < 0.05).

**Table 4:** Free amino acid contents of *cheonggukjang* according to the addition ratio of mountain-cultivated ginseng.

Indexs ^1^ (mg/100 g)	Addition Ratio of Mountain-Cultivated Ginseng (%)							
	Unfermented *Chenoggukjang*				Fermented *Chenoggukjang*			
	0	2.5	5.0	10	0	2.5	5.0	10
Non-essential amino acids								
Taurine	nd ^2^	nd	nd	2.78 ± 0.03a	nd	nd	nd	nd
Proline	nd	nd	nd	nd	1161.13 ± 25.25a	895.08 ± 18.14c	712.64 ± 17.11d	922.45 ± 20.11b
Aspartic acid	23.47 ± 0.27g	23.74 ± 0.29f	24.88 ± 0.25e	23.77 ± 0.25f	479.44 ± 7.88a	310.05 ± 6.45b	207.11 ± 4.17d	245.52 ± 6.61c
Serine	8.33 ± 0.13g	8.35 ± 0.11g	8.84 ± 0.09e	8.59 ± 0.10f	57.54 ± 1.81a	47.35 ± 0.58b	34.28 ± 0.74c	30.00 ± 0.80d
Aspartic acid-NH_2_	14.02 ± 0.18e	14.08 ± 0.18e	16.66 ± 0.17d	14.01 ± 0.18e	42.58 ± 1.03b	45.02 ± 0.44a	21.24 ± 0.51c	nd
Glutamic acid	65.90 ± 0.68e	60.97 ± 0.65f	61.81 ± 0.60f	54.68 ± 0.58g	2720.27 ± 35.11a	1796.58 ± 28.75c	1401.07 ± 20.11d	1960.25 ± 36.10b
Aminoadipic acid	5.12 ± 0.02e	4.03 ± 0.09f	4.51 ± 0.05f	3.15 ± 0.08g	266.20 ± 4.41d	421.62 ± 6.12c	462.98 ± 7.13a	438.82 ± 9.59b
Glycine	15.42 ± 0.30e	14.62 ± 0.18f	14.55 ± 0.16f	13.51 ± 0.15g	390.95 ± 4.52a	327.19 ± 5.72b	265.37 ± 4.55d	301.09 ± 7.11c
Alanine	40.21 ± 0.42e	38.20 ± 0.45g	39.23 ± 0.40f	33.78 ± 0.34h	905.70 ± 11.52d	1130.12 ± 18.21a	1020.89 ± 10.21c	1097.08 ± 21.17b
Citrulline	nd	nd	nd	nd	545.25 ± 8.44a	306.26 ± 5.22b	200.63 ± 6.11d	269.96 ± 5.11c
α-aminobutyric aicd	nd	1.47 ± 0.01e	1.57 ± 0.02e	nd	9.49 ± 0.12a	2.39 ± 0.02c	1.99 ± 0.05d	3.30 ± 0.03b
Cystine	21.40 ± 0.35d	20.54 ± 0.35d	18.30 ± 0.20e	17.35 ± 0.20e	102.34 ± 2.15a	82.51 ± 0.93b	64.56 ± 1.15c	64.25 ± 1.04c
Tyrosine	17.75 ± 0.32e	16.56 ± 0.19f	16.49 ± 0.18f	15.89 ± 0.18g	868.52 ± 15.95a	805.91 ± 11.56b	707.19 ± 15.17d	734.37 ± 11.14c
β-Alanine	3.19 ± 0.03f	3.21 ± 0.05f	3.44 ± 0.03e	3.43 ± 0.03d	16.36 ± 0.35c	20.65 ± 0.21b	21.98 ± 0.22a	20.96 ± 0.21b
β-aminoisobutyric acid	1.80 ± 0.01e	1.43 ± 0.01f	1.58 ± 0.02g	1.67 ± 0.02f	236.09 ± 3.89c	250.54 ± 5.11a	228.09 ± 5.88d	246.77 ± 8.89b
γ-aminobutyric acid	36.11 ± 0.37d	38.14 ± 0.42c	41.64 ± 0.48b	45.71 ± 0.49a	8.78 ± 0.09h	15.42 ± 0.24g	21.32 ± 0.21f	24.86 ± 0.25e
Aminoetahnol	5.97 ± 0.08d	3.24 ± 0.03e	3.03 ± 0.03e	3.11 ± 0.03e	10.26 ± 0.15c	1.56 ± 0.02f	10.89 ± 0.15b	14.19 ± 0.25a
Hydroxylysine	9.66 ± 0.11d	9.51 ± 0.11e	9.70 ± 0.10d	9.53 ± 0.10e	21.65 ± 0.22c	25.49 ± 0.28a	23.68 ± 0.31b	25.16 ± 0.31a
Ornithine	nd	nd	nd	nd	572.91 ± 8.87c	600.09 ± 6.85b	606.71 ± 6.57b	681.77 ± 7.82a
Anserine	nd	nd	nd	nd	39.40 ± 0.91a	nd	nd	nd
Arginine	190.41 ± 1.90d	206.05 ± 2.26c	244.14 ± 2.34a	221.16 ± 2.33b	14.59 ± 0.18e	9.12 ± 0.09f	9.65 ± 0.11f	nd
Total	458.76 ± 4.90h	464.14 ± 5.38g	510.37 ± 5.12e	472.12 ± 5.09f	8469.45 ± 132.85a	7092.95 ± 114.94b	6022.27 ± 100.46d	7080.8 ± 136.54c
Essential amino acids								
Threonine	6.82 ± 0.11f	6.99 ± 0.07f	6.96 ± 0.07f	7.29 ± 0.07e	435.24 ± 9.75a	400.59 ± 7.52b	367.59 ± 8.18d	385.84 ± 8.16c
Valine	13.85 ± 0.18f	14.13 ± 0.17e	13.94 ± 0.18f	14.22 ± 0.17e	1006.66 ± 15.15a	911.04 ± 15.01b	788.20 ± 18.11d	802.19 ± 19.11c
Methionine	5.20 ± 0.08d	4.82 ± 0.05e	4.74 ± 0.05e	4.31 ± 0.04f	327.18 ± 5.11a	302.00 ± 7.12b	268.66 ± 5.87c	267.97 ± 5.48c
Isoleucine	5.00 ± 0.05f	5.28 ± 0.05f	5.08 ± 0.05f	5.66 ± 0.06e	864.91 ± 18.88a	799.15 ± 15.01b	654.64 ± 11.05c	635.13 ± 10.55d
Leucine	9.30 ± 0.11f	9.71 ± 0.10e	9.53 ± 0.15e	10.82 ± 0.11d	1494.15 ± 35.88a	1406.59 ± 21.71b	1218.38 ± 17.81c	1210.99 ± 25.01c
Phenylalanine	23.46 ± 0.29f	25.10 ± 0.28e	23.32 ± 0.28f	23.73 ± 0.30f	1108.06 ± 29.15a	1048.15 ± 15.58b	928.62 ± 10.19d	945.81 ± 11.06c
Lysine	10.50 ± 0.15f	10.84 ± 0.11e	10.00 ± 0.15f	10.55 ± 0.11f	1244.72 ± 25.48a	1102.12 ± 17.02b	943.42 ± 11.03d	1028.93 ± 12.09c
Histamine	3.88 ± 0.03f	3.83 ± 0.04g	5.49 ± 0.06e	3.15 ± 0.08g	380.74 ± 7.74b	338.81 ± 4.81a	280.29 ± 3.10d	308.31 ± 7.11c
Total	78.01 ± 1.00f	80.7 ± 0.87e	79.06 ± 0.99e	79.73 ± 0.94e	6861.66 ± 147.14a	6308.45 ± 103.78b	5449.8 ± 85.34d	5585.17 ± 98.57c
Total amino acids	536.77 ± 5.90g	544.84 ± 6.25g	589.43 ± 6.11e	551.85 ± 6.03f	15,331.11 ± 279.99a	13,401.40 ± 218.72b	11,472.07 ± 185.80d	12,665.97 ± 235.11c
Ammonia	27.44 ± 0.25e	20.76 ± 0.21f	19.57 ± 0.20g	20.70 ± 0.21f	345.94 ± 3.46a	332.52 ± 3.33b	322.23 ± 3.22c	310.01 ± 3.10d

^1^ All values are presented as the mean ± standard deviation of pentaplicate determination. Means with different letters within a row are significantly different between samples for the same index (*p* < 0.05). ^2^ nd, not detected.

**Table 5:** Fatty acid contents of *cheonggukjang* according to the addition ratio of mountain-cultivated ginseng.

Index ^1^ (mg/100 g)	Addition Ratio of Mountain-Cultivated Ginseng (%)							
	Unfermented *Chenoggukjang*				Fermented *Chenoggukjang*			
	0	2.5	5.0	10	0	2.5	5.0	10
Saturated fatty acids								
Myristic acid (C14:0)	1.04±0.01e	2.45 ± 0.02b	nd ^2^	1.02 ± 0.01f	2.44 ± 0.02b	4.49 ± 0.05a	2.01 ± 0.03d	2.14 ± 0.02c
Palmitic acid (C16:0)	171.38 ± 2.51d	172.92 ± 2.13d	162.81 ± 3.39f	129.42 ± 5.07f	185.97 ± 3.68a	186.35 ± 4.06a	180.61 ± 4.01b	176.84 ± 3.58c
Stearic acid (C18:0)	64.87 ± 0.85c	63.56 ± 0.81f	62.52 ± 0.63f	48.30 ± 0.95e	67.55 ± 1.08a	66.54 ± 0.97b	63.28 ± 0.93f	64.29 ± 1.14d
Arachidic acid (C20:0)	5.14 ± 0.06d	5.02 ± 0.05d	4.99 ± 0.05e	3.78 ± 0.06f	5.53 ± 0.06a	5.49 ± 0.07b	5.21 ± 0.08c	5.29 ± 0.10c
Behenic acid (C22:0)	13.26 ± 0.18e	7.25 ± 0.11f	6.86 ± 0.10f	5.07 ± 0.08f	29.84 ± 0.70a	19.00 ± 0.19d	27.41 ± 0.55b	23.37 ± 0.51c
Lignoceric acid (C24:0)	3.18 ± 0.03a	2.15 ± 0.02e	2.21 ± 0.02f	1.73 ± 0.02f	2.53 ± 0.03e	3.12 ± 0.02b	2.93 ± 0.03d	3.02 ± 0.03c
Total	258.87 ± 3.64d	253.35 ± 3.14d	239.39 ± 4.19f	189.32 ± 6.19f	293.86 ± 5.57a	284.99 ± 5.36b	281.45 ± 5.63b	274.9 ± 5.38c
Unsaturated fatty acids								
Palmitoleic acid (C16:1)	1.04 ± 0.01f	2.96 ± 0.03c	1.14 ± 0.01f	0.95 ± 0.01f	2.07 ± 0.02d	4.59 ± 0.05a	1.95 ± 0.03e	2.45 ± 0.02b
Elaidic acid (C18:1t)	5.75 ± 0.06e	1.26 ± 0.01f	nd ^2^	nd	19.17 ± 0.38a	10.86 ± 0.30d	15.40 ± 0.18b	14.38 ± 0.18c
Oleic acid (C18:1c)	319.88 ± 7.02c	325.63 ± 7.86b	337.74 ± 7.48a	244.59 ± 6.25	297.49 ± 6.01d	340.89 ± 11.05a	281.55 ± 5.21f	290.85 ± 5.41e
Linolelaidic acid (18:2t)	5.51 ± 0.06c	0.92 ± 0.00d	nd	nd	0.49 ± 0.00e	nd	9.52 ± 0.10a	9.38 ± 0.09b
Linoleic acid (C18:2c)	700.32 ± 12.21c	754.74 ± 13.35a	715.75 ± 15.14b	588.46 ± 15.81f	603.13 ± 14.03e	659.78 ± 18.60d	602.09 ± 15.44e	608.77 ± 10.01e
ɤ-Linolenic acid(C18:3n6)	nd	nd	nd	nd	1.49 ± 0.01a	1.21 ± 0.01c	1.18 ± 0.01b	1.21 ± 0.01c
Eicosenic acid (C20:1)	2.53 ± 0.03e	3.21 ± 0.03b	2.83 ± 0.03c	2.17 ± 0.02f	2.77 ± 0.03d	3.87 ± 0.02a	2.35 ± 0.03e	2.53 ± 0.03e
α-Linolenic acid (C18:3n3)	120.81 ± 8.19c	134.08 ± 3.84a	125.36 ± 3.59b	105.04 ± 5.05e	99.94 ± 2.05e	112.83 ± 2.93d	91.92 ± 1.22f	99.73 ± 1.50e
Eicosadienoic acid (C20:2)	13.21 ± 0.23d	2.85 ± 0.01e	1.69 ± 0.02f	1.03 ± 0.00f	35.04 ± 0.81a	23.30 ± 0.35c	34.57 ± 0.48a	30.26 ± 0.40b
Eicosatrienoic acid (C20:3n6)	nd	1.48 ± 0.01b	nd	nd	nd	nd	nd	18.71 ± 0.21a
Erucic acid (C22:1n9)	nd	1.86 ± 0.02b	nd	nd	0.54 ± 0.00c	2.66 ± 0.02a	nd	nd
Arachidonic acid (C20:4n6)	nd	nd	nd	nd	0.95 ± 0.01b	1.42 ± 0.01a	1.49 ± 0.01a	1.53 ± 0.02a
Eicosapentaenoic acid (C20:5n3)	nd	nd	nd	nd	0.90 ± 0.01b	1.37 ± 0.01a	0.77 ± 0.01c	0.63 ± 0.01d
Nervonic acid (C24:1n9)	1.03 ± 0.01e	nd	nd	nd	1.83 ± 0.02a	1.24 ± 0.01d	1.79 ± 0.02b	1.61 ± 0.02c
Total	1171.13 ± 27.76c	1228.99 ± 25.16a	1184.51 ± 26.27b	942.9 ± 22.09f	1065.81 ± 23.38f	1164.02 ± 33.36	1044.58 ± 22.74g	1082.04 ± 17.55e
Total fatty acids	1430.0 ± 31.40c	1482.34 ± 28.30a	1424.9 ± 30.46d	1132.22 ± 28.28g	1359.67 ± 28.95e	1449.01 ± 38.72b	1326.03 ± 28.37f	1356.99 ± 22.93e

^1^ All values are presented as the mean ± standard deviation of pentaplicate determination. Means with different letters within a row are significantly different between samples for the same index (*p* < 0.05). ^2^ nd, not detected.

**Table 6:** Isoflavone and ginsenoside contents of *cheonggukjang* according to the addition ratio of mountain-cultivated ginseng.

Contents ^1^ (μg/g d.w.)	Addition Ratio of Mountain-Cultivated Ginseng (%)							
	Unfermented *Chenoggukjang*				Fermented *Chenoggukjang*			
	0	2.5	5.0	10	0	2.5	5.0	10
Isoflavones								
Daidzin	669.44 ± 13.14a	659.43 ± 13.19a	633.50 ± 12.67b	590.05 ± 10.25c	138.14 ± 3.45d	140.44 ± 2.91d	135.52 ± 2.54d	121.71 ± 2.44e
Glycitin	309.76 ± 6.21a	306.36 ± 6.13a	317.94 ± 6.36a	285.72 ± 5.71b	255.14 ± 4.21c	194.13 ± 3.54d	194.88 ± 3.88d	168.94 ± 3.12e
Genistin	847.44 ± 14.25a	856.8 ± 17.14a	804.36 ± 14.22b	739.92 ± 14.80c	93.68 ± 1.87d	94.48 ± 1.87d	85.95 ± 1.68e	95.83 ± 2.00d
Daidzein	57.65 ± 1.15e	51.26 ± 1.03f	48.06 ± 0.96g	48.34 ± 0.97g	613.53 ± 12.25a	575.99 ± 11.52b	545.13 ± 10.88c	483.17 ± 9.55d
Glycitein	9.71 ± 0.19d	nd ^2^	nd	nd	87.06 ± 1.74b	93.24 ± 1.86a	95.69 ± 2.01a	82.52 ± 1.70c
Genistein	48.81 ± 0.98d	45.54 ± 0.91d	45.39 ± 0.88d	45.14 ± 0.90d	354.41 ± 7.09c	421.7 ± 7.78a	378.41 ± 7.88b	343.62 ± 6.54c
Total	1942.81 ± 35.92a	1919.39 ± 38.39a	1849.25 ± 35.09b	1709.17 ± 32.63c	1540.96 ± 30.61d	1519.98 ± 29.48e	1435.58 ± 28.87f	1295.79 ± 25.35g
Ginsenosides								
Ginsenoside Rg1	nd	nd	nd	65.64 ± 1.51a	nd	nd	33.28 ± 0.74c	45.52 ± 1.01b
Ginsenoside Re	nd	nd	99.35 ± 2.01b	258.33 ± 6.14a	nd	nd	33.60 ± 0.77d	58.85 ± 1.24c
Ginsenoside Rf	nd	19.56 ± 0.39d	31.64 ± 0.66b	88.94 ± 1.88a	nd	nd	nd	22.01 ± 0.38c
Ginsenoside Rg2	nd	129.51 ± 2.11d	187.22 ± 3.24c	360.83 ± 7.54a	nd	nd	nd	280.23 ± 5.77b
Ginsenoside F1	nd	nd	nd	54.09 ± 1.25a	nd	nd	nd	12.15 ± 0.28b
Protopanaxtriol	nd	37.00 ± 0.84c	36.33 ± 0.87c	39.93 ± 0.91b	nd	29.25 ± 0.61d	30.06 ± 0.65d	56.79 ± 1.45a
Ginsenoside Rb2	nd	nd	183.40 ± 3.77b	294.09 ± 5.99a	nd	nd	nd	44.57 ± 1.01b
Ginsenoside Rd	nd	nd	nd	nd	nd	nd	18.08 ± 0.37a	nd
Ginsenoside Rd2	nd	nd	nd	327.40 ± 6.75a	nd	96.27 ± 2.11c	94.63 ± 1.99c	146.20 ± 3.22b
Ginsenoside F2	nd	66.62 ± 1.64f	110.48 ± 2.54e	165.61 ± 3.48d	nd	200.35 ± 3.58c	233.68 ± 4.54b	298.72 ± 6.10a
Ginsenoside Rg3	nd	56.51 ± 1.03e	65.56 ± 1.01d	96.05 ± 2.00b	nd	89.43 ± 1.87c	94.71 ± 1.99b	166.90 ± 3.84a
Compound K	nd	28.54 ± 0.61f	41.63 ± 0.90e	96.23 ± 2.15c	nd	69.43 ± 1.48d	150.72 ± 3.12b	231.33 ± 4.88a
Ginsenoside Rh2	nd	nd	nd	28.60 ± 0.61c	nd	24.58 ± 0.51d	33.94 ± 0.71b	82.32 ± 1.97a
Protopanaxdiol	nd	706.23 ± 15.11d	811.50 ± 16.55c	843.07 ± 17.01b	nd	806.48 ± 16.24c	840.29 ± 17.11b	1035.15 ± 21.45a
Total	nd	1043.97 ± 21.73e	1567.11 ± 31.55c	2718.81 ± 57.22a	nd	1315.79 ± 26.40d	1562.99 ± 31.99c	2480.74 ± 52.60b

^1^ All values are presented as the mean ± standard deviation of pentaplicate determination. Means with different letters within a row are significantly different between samples for the same index (*p* < 0.05). ^2^ nd, not detected.

## Data Availability

The original contributions presented in the study are included in the article; further inquiries can be directed to the corresponding author.

## Supplementary Materials

The following supporting information can be downloaded at: https://www.mdpi.com/article/10.3390/foods13193155/s1, Table S1: Primer for PCR amplification of 16S rRNA*, rec*A, and *gyr*B; Table S2: Physiological and biochemical characteristics of the fermented soybean strains with IDCK30 and IDCK40; Table S3: The cell wall fatty acid compositions of the fermented soybean strains with IDCK30 and IDCK40; Figure S1: The images of *cheonggukjang* according to the addition ratio of mountain cultivated ginseng; Figure S2: Nucleotide sequence of 16S rRNA gene from *Bacillus licheniformis* IDCK30; Figure S3: Nucleotide sequence of 16S rRNA gene from *Bacillus subtilis* IDCK40; Figure S4: Nucleotide sequence of *rec*A gene from *Bacillus licheniformis* IDCK30; Figure S5: Nucleotide sequence of *rec*A gene from *Bacillus subtilis* IDCK40; Figure S6: Nucleotide sequence of *gyr*B gene from *Bacillus licheniformis* IDCK30; Figure S7: Nucleotide sequence of *gyr*B gene from *Bacillus subtilis* IDCK40.

## References

[1] Lee J.H., Kim S.C., Lee H.Y., Cho D.Y., Jung J.G., Kang D.W., Kang S.S., Cho K.M. (2021). Changes in nutritional compositions of processed mountain-cultivated ginseng sprouts (*Panax ginseng*) and screening for their antioxidant and anti-inflammatory properties. J. Funct. Food..

[2] Xu X.F., Cheng X.L., Lin Q.H., Li S.S., Jia Z., Han T., Lin R.C., Wang D., Wei F., Li X.R. (2016). Identification of mountain-cultivated ginseng and cultivated ginseng using UPLC/oa-TOF MSE with a multivariate statistical sample-profiling strategy. J. Ginseng Res..

[3] Tran T.H.M., Puja A.M., Kim H., Kim Y.J. (2022). Nanoemulsions prepared from mountain ginseng-mediated gold nanoparticles and silydianin increase the anti-inflammatory effects by regulating NF- κB and MAOK signaling pathways. Biomater. Adv..

[4] Eom S.J., Hwang J.E., Kim H.S., Kim K.T., Paik H.D. (2018). Anti-inflammatory and cytotoxic effects of ginseng extract bioconverted by *Leuconostoc mesenteroides* KCCM 12010P isolated from kimchi. Int. J. Food Sci. Technol..

[5] Abdelfattah-Hassan A., Shalaby S.I., Khater S.I., El-Shetry E.S., El Fadil H.A., Elsayed S.A. (2019). *Panax ginseng* is superior to vitamin E as a hepatoprotector against cyclophosphamide-induced liver damage. Complement. Ther. Med..

[6] Park S.H., Chung S., Chung M.Y., Choi H.K., Hwang J.T., Park J.H. (2022). Effects of *Panax ginseng* on hyperglycemia, hypertension, and hyperlipidemia: A systematic review and meta-analysis. J. Ginseng Res..

[7] Kim J.S., Yoo J.M., Park J.E., Kim J., Kim S.G., Seok Y.M., Son J.H., Kim H.J. (2021). Anti-angiogenic effect of mountain ginseng in vitro and in vivo: Comparison with farm-cultivated ginseng. Mol. Med. Rep..

[8] Kim C.K., Cho D.H., Lee K.S., Lee D.K., Park C.W., Kim W.G., Lee S.J., Ha K.S., Taeg O.G., Kwon Y.G. (2012). Ginseng berry extract prevents atherogenesis via anti-inflammatory action by upregulating phase II gene expression. Evid. Based Complement. Altern. Med..

[9] Park D.H., Han B., Shin M.-S., Hwang G.S. (2020). Enhanced Intestinal Immune Response in Mice after Oral Administration of Korea Red Ginseng-Derived Polysaccharide. Polymers.

[10] Chen X., Lu Y., Zhao A., Wu Y., Zhang Y., Yang X. (2022). Quantitative analysis for several nutrients and volatile components during fermentation of soybean by *Bacillus subtilis natto*. Food Chem..

[11] Lee D.H., Kim M.J., Ahn J., Lee S.H., Lee H.J., Kim J.H., Park S.H., Jang Y.J., Jung C.H. (2017). Nutrikinetics of Isoflavone Metabolites After Fermented Soybean Product (*Cheonggukjang*) Ingestion in Ovariectomized Mice. Mol. Nutr. Food Res..

[12] Kim S.H., Ko J., Kwon D.Y. (2023). Jang, Korean fermented soybean product, the result of endeavors of ancients for the best taste of Korean diet. J. Ethn. Food..

[13] Ghosh K., Kang H.S., Hyun W.B., Kim K.P. (2018). High prevalence of *Bacillus subtilis*-infecting bacteriophages in soybean-based fermented foods and its detrimental effects on the process and quality of *Cheonggukjang*. Food Microbiol..

[14] Kim I.-S., Hwang C.-W., Yang W.-S., Kim C.-H. (2021). Current Perspectives on the Physiological Activities of Fermented Soybean-Derived *Cheonggukjang*. Int. J. Mol. Sci..

[15] Kim J.H., Hwang C.E., Lee C.K., Lee J.H., Kim G.M., Jeong S.H., Shin J.H., Kim J.S., Cho K.M. (2014). Characteristics and antioxidant effect of garlic in the fermentation of *Cheonggukjang* by *Bacillus amyloliquefaciens* MJ1-4. J. Microbiol. Biotechnol..

[16] Hong S.C., Kwan D.J. (2011). Changes in quality characteristics of Cheongkukjang added with Deodeok. Korean J. Food Preserv..

[17] Choi E.J., Lee J.S., Chang H.B., Lee M.S., Jang H.D., Kwon Y.I. (2010). Changes in the functionality of Cheonggukjang during fermentation supplemented with *Angelica gigas*, *Rehmanniae Radix*, and Red ginseng. Microbiol. Biotechnol. Lett..

[18] Saitou N., Nei M. (1987). The neighbor-joining method: A new method for reconstructing phylogenetic trees. Mol. Biol. Evol..

[19] Mahaffee W.F., Kloepper J.W. (1997). Temporal changes in the bacterial communities of soil, rhizosphere, and endorhiza associated with field-grown cucumber (*Cucumis sativus* L.). Microb. Ecol..

[20] Seo H.R., Kim J.Y., Kim J.H., Park K.Y. (2009). Identification of *Bacillus cereus* in a Chungkukjang That Showed High Anticancer Effects Against AGS Human Gastric Adenocarcinoma Cells. J. Med. Food..

[21] Goldschmidt-Clermont E., Hochwartner O., Demarta A., Caminada A.P., Fery J. (2009). Outbreaks of an ulcerative and haemorrhagic disease in Arctic char *Salvelinus alpinus* caused by *Aeromonas salmonicida* subsp. smithia. Dis. Aquat. Org..

[22] Vargas-Bello-Pérez E., Pedersen N.C., Khushvakov J., Ye Y., Dhakal R., Hansen H.H., Ahrné L., Khakimov B. (2022). Effect of supplementing dairy goat diets with rapeseed oil or sunflower oil on performance, Milk composition, Milk fatty acid profile, and in vitro fermentation kinetics. Front. Vet. Sci..

[23] Wang P.-Y., Shuang F.-F., Yang J.-X., Jv Y.-X., Hu R.-Z., Chen T., Yao X.-H., Zhao W.-G., Liu L., Zhang D.-Y. (2022). A rapid and efficient method of microwave-assisted extraction and hydrolysis and automatic amino acid analyzer determination of 17 amino acids from mulberry leaves. Ind. Crops Prod..

[24] Kuligowski M., Pawłowska K., Jasińska-Kuligowska I., Nowak J. (2017). Isoflavone composition, polyphenols content and antioxidative activity of soybean seeds during tempeh fermentation. CyTA-J. Food.

[25] Kim J.-H., Shin J.-S., Kim W., Lee H., Baik M.-Y. (2023). Effects of Puffing, Acid, and High Hydrostatic Pressure Treatments on Ginsenoside Profile and Antioxidant Capacity of Mountain-Cultivated *Panax ginseng*. Foods.

[26] Nam Y.D., Yi S.H., Lim S.I. (2012). Bacterial diversity of *cheonggukjang*, a traditional Korean fermented food, analyzed by barcoded pyrosequencing. Food Control.

[27] Cho K.M., Hong S.Y., Math R.K., Lee J.H., Kambiranda D.M., Kim J.M., Islam S.M.A., Yun M.G., Cho J.J., Lim W.J. (2009). Biotransformation of phenolics (isoflavones, flavanols and phenolic acids) during the fermentation of *cheonggukjang* by *Bacillus pumilus* HY1. Food Chem..

[28] Cho K.M., Lee J.H., Yun H.D., Ahn B.Y., Kim H., Seo W.T. (2011). Changes in phytochemical constituents (isoflavones, flavanols, and phenolic acids) during *cheonggukjang* soybeans fermentation using potential probiotics *Bacillus subtilis* CS90. J. Food Compos. Anal..

[29] Shin E.C., Lee J.H., Hwang C.E., Lee B.W., Kim H.T., Ko J.M., Baek I.Y., Shin J.H., Nam S.H., Seo W.T. (2014). Enhancement of total phenolic and isoflavone-aglycone contents and antioxidant activities during *Cheonggukjang* fermentation of brown soybeans by the potential probiotic *Bacillus subtilis* CSY191. Food Sci. Biotechnol..

[30] Lee H.Y., Lee J.H., Shin E.-C., Cho D.Y., Jung J.G., Kim M.J., Jeong J.B., Kang D., Kang S.S., Cho K.M. (2022). Changes in Chemical Compositions and Antioxidant Activities from Fresh to Fermented Red Mountain-Cultivated Ginseng. Molecules.

[31] Kim S.Y., Kim H.E., Kim Y.S. (2017). The potentials of *Bacillus licheniformis* strains for inhibition of *B. cereus* growth and reduction of biogenic amines in *cheonggukjang* (Korean fermented unsalted soybean paste). Food Control.

[32] Diana M., Rafecas M., Arco C., Quilez J. (2014). Free amino acid profile of Spanish artisanal cheeses: Importance of gamma-aminobutyric acid (GABA) and ornithine content. J. Food Compos. Anal..

[33] Yu J.J., Park K.B., Kim S.G., Oh S.H. (2013). Expression, purification, and biochemical properties of arginase from *Bacillus subtilis* 168. J. Microbiol..

[34] Cho K.M., Lim H.J., Kim M.S., Kim D.S., Hwang C.E., Nam S.H., Joo O.S., Lee B.W., Kim J.K., Shin E.C. (2017). Time course effects of fermentation on fatty acid and volatile compound profiles of *Cheonggukjang* using new soybean cultivars. J. Food Drug Anal..

[35] Chung I.M., Kim J.K., Yang J.H., Lee J.H., Park S.K., Son N.Y., Kim S.H. (2017). Effects of soil type and organic fertilizers on fatty acids and vitamin E in Korean ginseng (*Panax ginseng* Meyer). Food Res. Int..

[36] Hwang C.E., Kim S.C., Lee J.H., Hong S.Y., Cho K.M. (2018). Enhanced biological effect of fermented soy-powder milk with *Lactobacillus* brevis increasing in γ-aminobutyric acid and isoflavone aglycone contents. J. Appl. Biol. Chem..

[37] Lee M.J., Lee J.M., Kim S., Kim H.J. (2019). Simultaneous analysis and measurement of uncertainty estimation of six isoflavones in Cheonggukjang by liquid chromatography-electrospray tandem mass spectrometry. Food Chem..

[38] Lu C., Li F., Yan X., Mao S., Zhang T. (2022). Effect of pulsed electric field on soybean isoflavone glycosides hydrolysis by β-glucosidase: Investigation on enzyme characteristics and assisted reaction. Food Chem..

[39] Cairns J.R.K., Esen A. (2010). β-Glucosidases. Cell Mol. Life Sci..

[40] Silvia L.H., Celeghini R.M., Chang Y.K. (2011). Effect of the fermentation of whole soybean flour on the conversion of isoflavones from glycosides to aglycones. Food Chem..

[41] Piao Y.Z., Eun J.B. (2020). Physicochemical characteristics and isoflavones content during manufacture of short-time fermented soybean product (*cheonggukjang*). J. Food Sci. Technol..

[42] Zhu H., Zhang R., Huang Z., Zhou J. (2023). Progress in the Conversion of Ginsenoside Rb1 into Minor Ginsenosides Using β-Glucosidases. Foods.

[43] Park C.S., Yoo M.H., Noh K.H., Oh D.K. (2010). Biotransformation of ginsenosides by hydrolyzing the sugar moieties of ginsenosides using microbial glycosidases. Appl. Microbiol. Biotechnol..

[44] Jang G.Y., Kim M.Y., Lee Y.J., Li M., Shin Y.S., Lee J.S., Jeong H.S. (2018). Influence of organic acids and heat treatment on ginsenoside conversion. J. Ginseng Res..

[45] Kim K.A., Jung I.H., Park S.H., Ahn Y.T., Huh C.S., Kim D.H. (2013). Comparative analysis of the gut microbiota in people with different levels of ginsenoside Rb1 degradation to compound K. PLoS ONE.

[46] Duan Z., Zhu C., Shi J., Fan D., Deng J., Fu R., Huang R., Fan C. (2018). High efficiency production of ginsenoside compound K by catalyzing ginsenoside Rb1 using snailase. Chin. J. Chem. Eng..

[47] Kim J.H., Yi Y.S., Kim M.Y., Cho J.Y. (2017). Role of ginsenosides, the main active components of *Panax ginseng*, in inflammatory responses and diseases. J. Ginseng Res..

[48] Bekhit A.E.A., Duncan A., Bah C.S.F., Ahmed I.A.M., Al-Juhaimi F.Y., Amin H.F. (2018). Impact of fermentation conditions on the physicochemical properties, fatty acid and cholesterol contents in salted-fermented hoki roe. Food Chem..

[49] Saraç N., Şen B. (2014). Antioxidant, mutagenic, antimutagenic activities, and phenolic compounds of *Liquidambar orientalis* Mill. var. orientalis. Ind. Crops Prod..

[50] Makhafola T.J., Elgorashi E.E., McGaw L.J., Verschaeve L., Eloff J.N. (2016). The correlation between antimutagenic activity and total phenolic content of extracts of 31 plant species with high antioxidant activity. BMC Complement. Altern. Med..

[51] Ramadan D.T., Ali M.A.M., Yahya S.M., El-Sayed W.M. (2019). Correlation between Antioxidant/Antimutagenic and Antiproliferative Activity of Some Phytochemicals. Anticancer Agents Med. Chem..

[52] Ali M.W., Shahzad R., Bilal S., Adhikari B., Kim I.D., Lee J.D., Lee I.J., Kim B.O., Shin D.H. (2018). Comparison of antioxidants potential, metabolites, and nutritional profiles of Korean fermented soybean (*Cheonggukjang*) with *Bacillus subtilis* KCTC 13241. J. Food Sci. Technol..

[53] Chai C., Ju H.K., Kim S.C., Park J.H., Lim J., Kwon S.W., Lee J. (2012). Determination of bioactive compounds in fermented soybean products using GC/MS and further investigation of correlation of their bioactivities. J. Chromatogr. B.

[54] Zhao B., Wang X., Liu H., Lv C., Lu J. (2020). Structural characterization and antioxidant activity of oligosaccharides from *Panax ginseng* C. A. Meyer. Int. J. Biol. Macromol..

[55] Sanjukta S., Rai A.K., Muhammed A., Jeyaram K., Talukdar N.C. (2015). Enhancement of antioxidant properties of two soybean varieties of Sikkim Himalayan region by proteolytic *Bacillus subtilis* fermentation. J. Funct. Foods.

