# Supplementary material for: Comparison in Bioactive Compounds and Antioxidant Activity of Cheonggukjang Containing Mountain-Cultivated Ginseng Using Two Bacillus Genus
Source: Foods. 2024 Oct 3;13(19):3155. doi: 10.3390/foods13193155 (PMC11475840; doi:10.3390/foods13193155)
Supplement: Supplementary file 1 [file foods-13-03155-s001.zip › foods-3197079-supplementary.pdf]

Supplementary data

Table S1. Primer for PCR amplification of 16S rRNA, *rec*A, and *gyr*B

Genes	Isolates	Primer sequences (5′ → 3′)
16S rRNA	16SrRNA-F	CGGAGAGTTTGATCCTGG
	16SrRNA-R	TACGGCTACCTTACGAC
*rec*A	*rec*A-F	TGAGTGATCGTCAGGCAGCCTTAG
	*rec*A-R	CYTBRGATAAGARTACCAWGMACCGC
*gyr*B	*gyr*B-F	GAAGTCATCATGACCGTTCTGCAYGCNGGNGGNAARTTYGA
	*gyr*B-R	AGCAGGGTACGGATGTGCGAGCCRTCNACRTCNGCRTCNGTCAT

Table S2. Physiological and biochemical characteristics of the fermented soybean strains with IDCK30 and IDCK40

Characteristics	Isolates / Reaction[1)]		Characteristics	Isolates / Reaction	
	IDCK 30	IDCK 40		IDCK 30	IDCK 40
**Morphology**			Dulcitol	-	-
Shape	Rod	Rod	Inositol	-	+
Gram stain	+	+	Mannitol	+	-
Cell dimension (μm)	1.5×0.5	1.4×0.6	Sorbitol	-	+
Flagellation	+	+	α-Methyl-D-mannoside	-	-
Endospore	+	+	α-Meyhyl-D-glucoside	+	+
**Physiology**			*N*-Acetyl glucosamine	+	-
Aerobic growth	+	+	Amygdaline	+	-
pH	3-11	3-11	Arbutine	+	-
Temperature (℃)	10 to 55	10 to 55	Esculine	+	+
NaCl (%)	<15	<15	Salicine	+	-
**Hydrolysis**			Cellobiose	+	-
Starch	+	+	Maltose	+	+
Cellulose	+	+	Lactose	-	-
Xylan	+	+	Melibiose	-	-
Mannan	-	+	Saccharose	+	+
Tributyrin	-	+	Trehalose	+	+
Skim milk	-	+	Inuline	-	+
**Carbohydrates**			Melezitose	-	-
Glycerol	+	-	D-Raffinose	-	+
Ertyhritol	-	-	Amidon	-	+
D-Arabinose	-	-	Glycogene	-	+
L-Arbinose	+	+	Xylitol	-	-
Ribose	+	+	β-Gentiobiose	-	-
D-Xylose	-	+	D-Turanose	-	-
L-Xylose	-	-	D-Lyxose	-	-
Adonitol	-	-	D-Tagatose	+	-
β-Methylxyloside	-	-	D-Fucose	-	-
Galactose	-	-	L-Fucose	-	-
D-Glucose	+	+	D-Arabitol	-	-
D-Fructose	+	+	S-Arabitol	-	-
D-Mannose	+	+	Gluconate	-	-
L-sorbose	-	-	2 ceto-gluconatd	-	-
Rhamnose	-	-	5 ceto-gluconate	-	-

[1)]Symbols: +, positive reaction; -, negative reaction.

Table S3. The cell wall fatty acid compositions of the fermented soybean strains with IDCK30 and IDCK40

Fatty acids[1)] (%)	Isolates	
	IDCK 30	IDCK 40
**Saturated fatty acids**		
C12:0	0.88	1.34
C14:0	0.69	nd[2)]
C16:0	7.48	3.83
C18:0	1.32	1.24
**Unsaturated fatty acids**		
C16:1 *w*7*c* alcohol	nd	0.52
**Branched fatty acids**		
iso-C14:0	1.64	3.18
iso-C15:0	24.75	12.70
anteiso-C15:0	32.15	47.31
iso-C16:0	7.27	9.61
iso-C17:0	10.02	7.71
anteiso-C17:0	13.78	12.00
iso-C17:1 *w*10*c*	nd	0.56

[1)]Fatty acids that be sperated by GC with the Microbial Identification (MIDI).
[2)]nd, not detected.

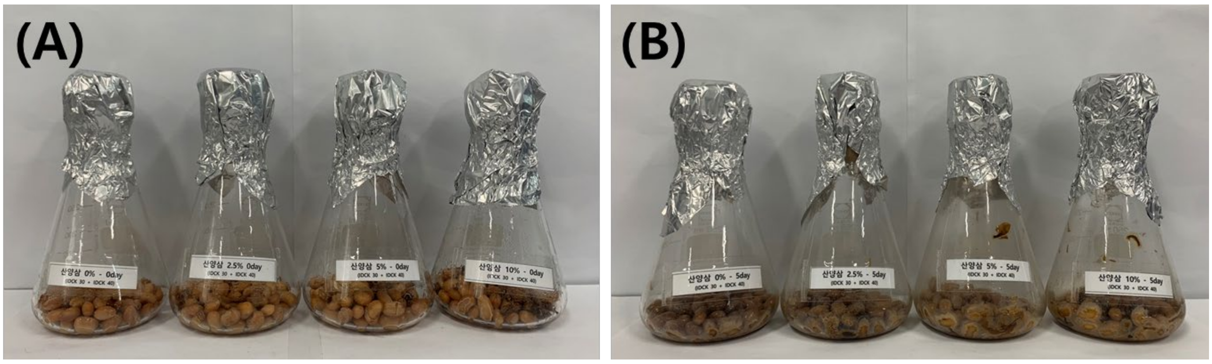

Figure S1. The images of *cheonggukjang* according to the addition ratio of mountain cultivated ginseng. (A) 0, 2.5, 5, and 10% MCG addition unfermented cheonggukjang. (B) 0, 2.5, 5, and 10% MCG addition fermented cheonggukjang.

```
   1 GCCTCTCAAA CTAGGACCGA GTCCTGCTTG CGACCGCCGC ACGGATTATG TACGTTCAGC
  61 TCGCCTGGCT GCCCTCGAAC GAGGGAATCC AGTCGCCGCC TGCCCACTCA TTGTGCACCC
 121 ATTGGACGGA CATTCTGACC CTATTGAGGC CCTTTGGCCC CGATTATGGC CTACGAACTA
 181 ACTTGGCGTA CCAAGTTAGT ATTTTCCACC GAAAATCGAT GGTGAATGTC TACCTGGGCG
 241 CCGCGTAATC GATCAACCAC TCCATTGCCG AGTGGTTCCG CTGCTACGCA TCGGCTGGAC
 301 TCTCCCACTA GCCGGTGTGA CCCTGACTCT GTGCCGGGTC TGAGGATGCC CTCCGTCGTC
 361 ATCCCTTAGA AGGCGTTACC TGCTTTCAGA CTGCCTCGTT GCGGCGCACT CACTACTTCC
 421 AAAAGCCTAG CATTTTGAGA CAACAATCCC TTCTTGTTCA TGGCAAGCTT ATCCCGCCAT
 481 GGAACTGCCA TGGATTGGTC TTTCGGTGCC GATTGATGCA CGGTCGTCGG CGCCATTATG
 541 CATCCACCGT TCGCAACAGG CCTTAATAAC CCGCATTTCG CGCGCGTCCG CCAAAGAATT
 601 CAGACTACAC TTTCGGGGGC CGAGTTGGCC CCTCCCAGTA ACCTTTGACC CCTTGAACTC
 661 ACGTCTTCTC CTCTCACCTT AAGGTGCACA TCGCCACTTT ACGCATCTCT ACACCTCCTT
 721 GTGGTCACCG CTTCCGCTGA GAGACCAGAC ATTGACTGCG ACTCCGCGCT TTCGCACCCC
 781 TCGCTTGTCC TAATCTATGG GACCATCAGG TGCGGCATTT GCTACTCACG ATTCACAATC
 841 TCCCAAAGGC GGGAAATCAC GACGTCGTTT GCGTAATTCG TGAGGCGGAC CCCTCATGCC
 901 AGCGTTCTGA CTTTGAGTTT CCTTAACTGC CCCCGGGCGT GTTCGCCACC TCGTACACCA
 961 AATTAAGCTT CGTTGCGCTT CTTGGAATGG TCCAGAACTG TAGGAGACTG TTGGGATCTC
1021 TATCCCGAAG GGGAAGCCCC CGTCTCACTG TCCACCACGT ACCAACAGCA GTCGAGCACA
1081 GCACTCTACA ACCCAATTCA GGGCGTTGCT CGCGTTGGGA ACTAGAATCA ACGGTCGTAA
1141 GTCAACCCGT GAGATTCCAC TGACGGCCAC TGTTTGGCCT CCTTCCACCC CTACTGCAGT
1201 TTAGTAGTAC GGGGAATACT GGACCCGATG TGTGCACGAT GTTACCCGTC TTGTTTCCCG
1261 TCGCTTCGGC GCTCCGATTC GGTTAGGGTG TTTAGACAAG AGTCAAGCCT AGCGTCAGAC
1321 GTTGAGCTGA CGCACTTCGA CCTTAGCGAT CATTAGCGCC TAGTCGTACG GCGCCACTTA
1381 TGCAAGGGCC CGGAACATGT GTGGCGGGCA GTGTGGTGCT CTCAAACATT GTGGGCTTCA
1441 GCCACTCCAT TGGAAAACCT CGGTCGGCGG CTTCCACCCT GTCTACTAAC CCCACTTCAG
1501 CATTGTTCCA TCGGCAT
```

Figure S2. Nucleotide sequence of 16S rRNA gene from *Bacillus licheniformis* IDCK30.

```
   1 CGGAGAGTTT GATCCTGGCT CAGGACGAAC GCTGGCGGCG TGCCTAATAC ATGCAAGTCG
  61 AGCGGACAGA TGGGAGCTTG CTCCCTGATG TTAGCGGCGG ACGGGTGAGT AACACGTGGG
 121 TAACCTGCCT GTAAGACTGG GATAACTCCG GGAAACCGGG GCTAATACCG GATGGTTGTT
 181 TGAATCGCAT GGTTCAAACA TAAAAGGTGG CTTCGGCTAC CACTTACAGA TGGACCCGCG
 241 GCGCATTAGC TAGTTGGTGA GGTAATGGCT CACCAAGGCG ACGATGCGTA GCCGACCTGA
 301 GAGGGTGATC GGCCACACTG GGACTGAGAC ACGGCCCAGA CTCCTACGGG AGGCAGCAGT
 361 AGGGAATCTT CCGCAATGGA CGAAAGTCTG ACGGAGCAAC GCCGCGTGAG TGATGAAGGT
 421 TTTCGGATCG TAAAGCTCTG TTGTTAGGGA AGAACAAGTA CCGTTCGAAT AGGGCGGTAC
 481 CTTGACGGTA CCTAACCAGA AAGCCACGGC TAACTACGTG CCAGCAGCCG CGGTAATACG
 541 TAGGTGGCAA GCGTTGTCCG GAATTATTGG GCGTAAAGGG CTCGCAGGCG GTTTCTTAAG
 601 TCTGATGTGA AAGCCCCCGG CTCAACCGGG GAGGGTCATT GGAAACTGGG GAACTTGAGT
 661 GCAGAAGAGG AGAGTGGAAT TCCACGTGTA GCGGTGAAAT GCGTAGAGAT GTGGAGGAAC
 721 ACCAGTGGCG AAGGCGACTC TCTGGTCTGT AACTGACGCT GAGGAGCGAA AGCGTGGGGA
 781 GCGAACAGGA TTAGATACCC TGGTAGTCCA CGCCGTAAAC GATGAGTGCT AAGTGTTAGG
 841 GGGTTTCCGC CCCTTAGTGC TGCAGCTAAC GCATTAAGCA CTCCGCCTGG GGAGTACGGT
 901 CGCAAGACTG AAACTCAAAG GAATTGACGG GGGCCCGCAC AAGCGGTGGA GCATGTGGTT
 961 TAATTCGAAG CAACGCGAAG AACCTTACCA GGTCTTGACA TCCTCTGACA ATCCTAGAGA
1021 TAGGACGTCC CCTTCGGGGG CAGAGTGACA GGTGGTGCAT GGTTGTCGTC AGCTCGTGTC
1081 GTGAGATGTT GGGTTAAGTC CCGCAACGAG CGCAACCCTT GATCTTAGTT GCCAGCATTC
1141 AGTTGGGCAC TCTAAGGTGA CTGCCGGTGA CAAACCGGAG GAAGGTGGGG ATGACGTCAA
1201 ATCATCATGC CCCTTATGAC CTGGGCTACG CACGTGCTAC AATGGACAGA ACAAAGGGCA
1261 GCGAAACCGC GAGGTTAAGC CAATCCCACA AATCTGTTCT CAGTTCGGAT CGCAGTCTGC
1321 AACTCGACTG CGTGAAGCTG GAATCGCTAG TAATCGCGGA TCAGCATGCC GCGGTGAATA
1381 CGTTCCCGGG CCTTGTACAC ACCGCCCGTC ACACCACGAG AGTTTGTAAC ACCCGAAGTC
1441 GGTGAGGTAA CCTTTTAGGA GCCAGCCGCC GAAGGTGGGA CAGATGATTG GGTGAAGTC
1501 GTAACAAGGT AGCCGTA
```

Figure S3. Nucleotide sequence of 16S rRNA gene from *Bacillus subtilis* IDCK40.

```
  1 CCTGAGATAA GAGTACCATG CACCGCTCTT TTGAACGATG TCAAGCTCTG TTCCGAGGTC
 61 GATGATTTCC CCTTCTTTTG AAATTCCTTC CCCGTACATA ATATCCACTT CGGCTGTCCG
121 GAATGGAGGT GCCACTTTGT TTTTCACGAC TTTGATTTTC GTCTTGTTCC CCATGACGTC
181 GTTGCCTTGT TTCAGCTGCT CTGCGCGGCG CACTTCAAGG CGGACAGAAG AGTAGAATTT
241 CAGCGCTCTT CCGCCTGGCG TCGTCTCAGG ATTTCCAAAC ATGACACCGA CTTTTTCACG
301 AATCTGGTTG ATAAAGATCG CGATGGTCTT CGATTTATTG ATCGCTCCGG AAAGCTTGCG
361 AAGCGCCTGA GACATCAGTC TGGCCTGCAA ACCGACGTGG GAATCCCCCA TATCTCCTTC
421 GATTTCAGCT TTCGGCACAA GCGCTGCTAC AGAGTCGATG ACAACGATAT CCACCGCTCC
481 GCTTCTGACA AGGGCTTCAG CGATTTCGAG CGCCTGCTCG CCCGTATCAG GCTGTGACAG
541 CAAAAGCTCA TCAATGTTGA CGCCCAGCTT TTGTGCATAG ACGGGATCAA GCGCGTGTTC
601 GGCGTCGATG AACGCCGCTT GTCCGCCCTG CTGCTGAACT TCGGCAATCG CATGAAGCGC
661 CACCGTCGTT TTACCGGAGC TTTCAGGCCC GTATACTTCA ATAATCCGGC CGCGCGGGTA
721 TCCGCCCACT CCAAGAGCCG CATCGAGCGC TAAAGAACCG CTCGGAACTG TTGAAATTCT
781 CGTTTCAGTT TGTTCGCCGA GTTTCATAAT CGAACCTTTA CCAAACTGCT TTTCTATTTG
841 TTTAAGCGCC ATATCTAAGG CTGCCTGACG ATCACTCA
```

Figure S4. Nucleotide sequence of *rec*A gene from *Bacillus licheniformis* IDCK30.

```
  1 CCTTGGATAA GAGTACCATG AACCGCTTTT TTGCACGATA TCAAGTTCAG TTCCTAGATC
 61 AATGATTTCG CCTTCTTTTG AAATGCCTTC TCCATACATA ATGTCAACCT CGGCTGTACG
121 GAACGGCGGA GCCACCTTGT TTTTCACGAC TTTGATTTTC GTTTTGTTCC CCATTATGTC
181 GTTGCCTTGT TTCAGCTGTT CAGCACGGCG CACTTCAAGA CGCACGGAAG AGTAGAATTT
241 CAACGCACGG CCGCCAGGAG TTGTTTCCGG GTTCCCGAAC ATAACGCCGA CTTTTTCACG
301 AATTTGGTTA ATGAAAATCG CGATTGTCTT CGATTTGTTA ATGGCCCCTG AAAGCTTACG
361 AAGCGCTTGA GACATTAAGC GTGCTTGTAA ACCGACATGA GAATCTCCCA TGTCGCCTTC
421 AATTTCCGCT TTCGGAACGA GAGCGGCTAC AGAGTCGACA ACGACAATGT CAACTGCCCC
481 GCTTCGAACC AATGCTTCCG CAATTTCAAG CGCCTGCTCG CCTGTGTCAG GCTGAGACAG
541 TAAAAGCTCC TCGATGTTAA CACCGAGCTT TTGCGCGTAT ACCGGATCTA ACGCATGCTC
601 CGCATCGATA AACGCGGCTT GTCCGCCCTG CTGCTGAACT TCAGCAATCG CATGAAGCGC
661 CACAGTTGTT TTACCTGAGC TTTCAGGACC GTATACTTCA ATAATCCGTC CGCGAGGATA
721 TCCGCCAATT CCCAGTGCTG TATCAAGAGC GAGGGAGCCG CTTGGGACAG TAGAAATTCT
781 TGTATCTGTC TTTTCTCCCA GTTTCATAAT GGAACCTTTG CCGAACTGGT TTTTCTATTT
841 GTTTAAGAGC CATATCTAAG GCTGCCTGAC GATCACTCA
```

Figure S5. Nucleotide sequence of *rec*A gene from *Bacillus subtilis* IDCK40.

```
   1 AGCAGGGTAC GGATGTGCGA GCCATCTACA TCAGCGTCTG TCATAATCAC GACTTTGTGG
  61 TAGCGGGCTT TTTCAAGGTT GAAATCTTCC CCGATTCCGG TGCCAAGGGC GGTGATCATA
 121 GAACGAACCT CATTGTTGGA CAATATTTTG TCCAGGCGGG CTTTTTCGAC GTTCAAAATT
 181 TTCCCTCTCA AAGGCAAAAT TGCTTGGAAA TGACGGTCGC GGCCCTGTTT TGCCGATCCG
 241 CCCGCAGAGT CACCCTCAAC GATGTAAAGT TCGGAAATCG TCGGGTCTTT AGAAGAACAG
 301 TCAGCAAGTT TCCCCGGCAG ATTGGACACT TCAAGGGCGC TTTTTCTGCG CGTCAATTCG
 361 CGTGCTTTCT TTGCAGCCAT CCGTGCTCTG GCGGCCATAA CCCCTTTTTC AACGATTTTT
 421 TTCGCTGAAT CCGGGTTTTC TAGCAGAAAC TTTTCAAGCG CTTCTGAAAA TAGCGCATCT
 481 GTTATCGTAC GCGCTTCTGA GTTGCCGAGC TTTGTTTTCG TCTGCCCTTC AAATTGAGGA
 541 TCCGGGTGCT TGATTGAAAT AATCGCTGTC AAACCTTCCC GGACGTCTTC TCCGCTTAAG
 601 TTCGGATCGC TTTCTTTGAA TACGCCGTTT CTTCTCGCGT AATCATTGAT GACCCTCGTC
 661 AAACCGGTCT TAAAGCCGGC TTCATGGGTT CCGCCTTCAT ACGTATGAAT GTTGTTAGCA
 721 AATGAATAAA TGTTGCTTGT ATAGCTGTCA TTGTATTGAA GAGCCACCTC GACTGTAATG
 781 CCGTCTTTGG ATCCTTCAAT ATAGACCGGC TCTTCATGAA TAACTTCCCG CGAACGGTTC
 841 AAGTGTTCAA CATAGCTTTT AATACCGCCT TCATAGCAGT ATTCATTCTT GCGTTCTTTT
 901 CCCTCTCGCT TGTCTTCGAT CGTGATTTTG ACGCCTTTTG TCAAGAAAGC GAGTTCGCGG
 961 ACACGAGTGG CGAGCGTATC ATAGTCGTAT TCAGTCGTTT CCGTGAATAT TTCCGGATCA
1021 GGCTTGAAGT GTGTGGTCGT TCCCGTCACT TCCGTATCTC CAATGACTTT CAAATCAGCT
1081 TTCGGGACGC CACGTTCAAA TTCCTGATAA TGGATTTTTC CATCTCTGTA AACCGTTACA
1141 TCCAGCTCGG TTGAAAGGGC GTTAACAACA GAAGCACCGA CGCCGTGCAA ACCGCCCGAG
1201 ACTTTATATC CGCTTCCGTC GAACTTCCCG CCAGCATGCA GAACGGTTCA TGATGACTTC
```

Figure S6. Nucleotide sequence of *gyr*B gene from *Bacillus licheniformis* IDCK30.

```
   1 AGCAGGGTAC GGATGTGCGA GCCGTCAACG TCTGCATCAG TCATAATGAC AACTTTGTGG
  61 TAACGGGCTT TCTCAAGGTT GAAGTCTTCC CCAATACCTG TGCCGAGCGC TGTGATCATA
 121 GAGCGAACTT CGTTGTTAGA AAGGATTTTA TCCAGTCTGG CCCTTTCAAC GTTTAGGATT
 181 TTACCTCTAA GCGGCAAAAT GGCTTGGAAA TGTCTGTCGC GTCCTTGTTT AGCAGATCCT
 241 CCGGCAGAGT CACCCTCTAC GATATATAAC TCGGAGATGC TCGGATCTTT TGAAGAGCAG
 301 TCCGCTAACT TACCGGGCAG GTTTGAAATT TCCAAAGCAC TCTTACGACG TGTTAGTTCA
 361 CGGGCTTTTT TCGCAGCCAT TCTTGCTCTT GCCGCCATTA AGCCTTTATC GACAATTTTT
 421 TTGGCTGCAT CTGGATTTTC CAGCATAAAT GTTTCCATCG CCGTAGAAAA TAACGTATCG
 481 GTGATCGTCC GTGCTTCTGA GTTGCCCAGC TTTGTTTTCG TTTGGCCCTC AAACTGCGGA
 541 TCAGGGTGTT TGATTGAAAT AATCGCTGTC AGCCCTTCCC TTACGTCATC TCCGCTTAGG
 601 TTTGGATCAT TTTCTTTAAT AAGCCCTTTT TTTCTGGCGT AATCGTTGAT AACACGAGTC
 661 AGGCCCGTTT TGAAGCCAGC TTCATGGGTA CCGCCTTCGT ACGTGTTAAT GTTGTTTGTA
 721 AACGAGTAAA TGTTGCTTGT GTAGCTGTCA TTGTATTGCA AAGCCACTTC AACCGTAATG
 781 CCGTCCTTTT CGCCTTCAAT GTAAATCGGC TCTTCATGGA CAACCTCTTT AGAGCGGTTT
 841 AAATACTCTA CATAACTTTT AATTCCGCCT TCGTAATGGT ATTCATTTTT GCGCTCTTGT
 901 CCTTCACGTT TATCCTCAAT CGTGATGTTT ACGCCCTTTG TTAAAAAGGC TAATTCACGT
 961 ACGCGGTTGG CAAGCAGATC ATAATCATAC TCGGTTGTTT CTGAGAAAAT TTCAGGGTCC
1021 GGGACAAAAT GTGTCGTCGT TCCTGTATGA TCCGTTTCGC CAATGATTTC AAGGTCTGTA
1081 ACCGGAACTC CGCGTTTATA AGTTTGGCGG TGAATTTTAC CGTCACGGTG AACCGTCACA
1141 TCAAGCTCTG TTGATAGTGC GTTTACGACA GACGCACCTA CACCGTGTAA TCCTCCGGAT
1201 ACTTTATAGC CGCTTCCGTC GAACTTTCCT CCGGCGTGCA GAACGGTCAT GATGACTTC
```

Figure S7. Nucleotide sequence of *gyr*B gene from *Bacillus subtilis* IDCK40.
